# A scalable and cost-effective nanopore workflow for 16S rRNA microbiome profiling validated in a zebrafish gut model

**DOI:** 10.1099/mgen.0.001828

**Published:** 2026-09-29

**Authors:** Maria Juanpere-Borras, Jos Boekhorst, Peter van Baarlen, Sylvia Brugman, Adrià López Nadal

**Affiliations:** 1 Host-Microbe Interactomics Group, NetherlandsWageningen University and Research, Wageningen

**Keywords:** amplicon sequence variant (ASV), Illumina, microbiome profiling, multiplexing, Nanopore, zebrafish

## Abstract

Advancements in long-read sequencing technologies, such as those developed by Oxford Nanopore Technologies (ONT), have opened new possibilities for 16S rRNA gene profiling of microbial communities by enabling full-length sequencing. In this study, we optimized and validated a cost-effective, in-house ONT workflow for zebrafish gut microbiome analysis and compared its performance to the gold-standard Illumina NovaSeq platform. Using a PCR-based barcoding strategy, we successfully sequenced both the V3–V4 and V1–V9 regions of the 16S rRNA gene, achieving high read accuracy and balanced demultiplexing across samples. Our results demonstrate that ONT short-read (V3–V4) sequencing using the latest V14 chemistry and Dorado basecaller achieves resolution comparable to that of Illumina, supporting amplicon sequence variant classification. Rarefaction analysis confirmed that the sequencing depth (~240,000 reads/sample) was sufficient to capture microbial diversity in the zebrafish gut, a complex and taxonomically rich environment. Furthermore, taxonomic classifications and diversity metrics were consistent between platforms, supporting the reliability of ONT for microbiota profiling. While full-length (V1–V9) ONT sequencing yielded higher taxonomic richness through operational taxonomic units, it did not significantly improve species-level resolution, likely due to current database and bioinformatics limitations. Our custom library preparation protocol simplified multiplexing, reduced costs and improved scalability without compromising accuracy. Although 15 samples were conservatively multiplexed per flow cell in this study, the workflow has since been applied to larger-scale datasets with up to 75 samples per run and to additional amplicon targets, demonstrating its portability and adaptability across diverse biological contexts. These findings position ONT as a viable, scalable and field-deployable alternative to Illumina for microbiome research.

**Impact Statement**

High-throughput microbiome profiling remains constrained by sequencing costs, infrastructure requirements and limited flexibility in multiplexing strategies. Here, we present a scalable and cost-effective PCR-based Nanopore workflow that enables accurate 16S rRNA gene profiling using both amplicon sequence variant inference for short-read amplicons and OTU clustering for full-length sequencing. Beyond laboratory validation, this workflow has already been applied across diverse biological contexts, including gut microbiome analyses in zebrafish and bees, as well as vertebrate and plant metabarcoding of wildlife samples using 12S rRNA and ITS2 markers. These applications demonstrate its adaptability beyond bacterial 16S profiling and its suitability for field-based sequencing. By lowering technical and financial barriers while maintaining analytical robustness, this study provides a portable and broadly applicable framework for microbial and environmental genomics research.

## Data Summary

All processed sequencing data (FASTQ files) generated from both Illumina and ONT platforms are publicly available in the European Nucleotide Archive (ENA) under project accession number PRJEB96324.

The NanoSplit16S Nextflow workflow used for demultiplexing and preprocessing Nanopore 16S amplicon data is openly available at GitHub (https://github.com/MariaJuanpereBorras/NanoSplit16S) [1]. ASV inference for V3–V4 amplicon reads and subsequent taxonomic classification were performed in QIIME2 (v2023.2.0) using the DADA2 plugin and the silva 16S database (v138). For full-length V1–V9 amplicon reads, OTU-based clustering and taxonomic assignment were conducted using the ONT-AmpSeq workflow with default parameters as recommended by the authors [2]. The workflow and associated scripts are publicly accessible via GitHub (https://github.com/MathiasEskildsen/ONT-AmpSeq). All additional software tools and version numbers are specified in the Methods section.

## Introduction

Technological advancements in multi-omics approaches have significantly expanded our understanding of the microbiome, particularly in identifying its composition and elucidating its role in disease onset and development [3, 4]. The bacterial composition of the microbiome is commonly determined through partial sequencing of the 16S rRNA gene, known as 16S rRNA profiling. The 16S rRNA gene is present in all bacteria and, along its length (i.e. ~1,500 bp), contains nine variable regions (V1–V9) (Fig. 1), the sequence of which informs taxonomic origin [5–7]. Traditionally, the platform chosen for 16S rRNA sequencing is the Illumina sequencing platform, which provides the basecalling accuracy needed for correct taxonomic profiling of the microbiome [7, 8]. However, one major limitation of Illumina sequencing is its inability to produce reads longer than 500 bp, potentially limiting taxonomic resolution since these regions might not capture the necessary genetic variation to delineate between all bacterial species [6, 7, 9].

The recent development of next-generation long-read sequencing technologies, such as PacBio and nanopore sequencing as developed by Oxford Nanopore Technologies (ONT), offers the opportunity to sequence the full-length 16S rRNA gene, potentially improving taxonomic classification [6, 8, 9]. ONT technologies support in-house sequencing through customizable workflows and portable devices, allowing flexible deployment from laboratory to field settings and reducing time and cost relative to outsourced sequencing, albeit with an increased risk of contamination under field conditions [10–12]. The biggest drawback of ONT has traditionally been its lower basecalling accuracy, which is crucial for taxonomic classification. However, the latest updates to ONT chemistry and its Dorado basecaller model have raised sequencing accuracy to over 99% [12, 13], yet even a 1% error rate may introduce substantial sequencing noise for taxonomic classifications based on the full-length 16S rRNA gene, where fine-scale resolution is critical. Due to this sensitivity, we consider 16S rRNA gene profiling an ideal test case for evaluating the robustness of the ONT sequencing platform. Numerous studies have already compared ONT and Illumina platforms for 16S rRNA microbiome profiling, highlighting their respective strengths and limitations. However, some studies [14–16] have utilized earlier versions of ONT chemistry (e.g. V12), which have since undergone significant advancements. Similarly, advancements in basecalling software, such as the transition from Guppy to the more accurate and powerful Dorado, highlight the rapid evolution of Nanopore technology [17, 18], though previous studies have relied on earlier basecalling models. Additionally, while published comparisons focus on ONT’s full-length 16S rRNA versus Illumina’s short-read sequencing, a critical limitation often arises: full-length 16S rRNA reads can accumulate too many sequencing errors, constraining analyses to operational taxonomic units (OTUs) rather than amplicon sequence variants (ASVs) [19, 20].

In this study, we compare a tailored ONT sequencing workflow with the commonly used Illumina sequencing platform to assess microbiota composition in zebrafish, a widely used experimental animal model. We used a real biological case study to assess whether both sequencing technologies could detect the same changes in microbiota composition. In previous studies, Brugman and co-workers observed statistically significant and biologically relevant shifts in microbiota composition in zebrafish lacking adaptive immune cells (Rag1-deficient zebrafish) [21]. Further investigation into this shift suggested that chemokine Cxcl8a might be required for this microbial modification [22]. To investigate the role of Cxcl8a, a novel CRISPR-Cas9-generated *cxcl8a* deletion mutant was generated, and 16S rRNA profiling of the zebrafish gut microbiota in both WT and *cxcl8a* deletion mutants was conducted.

Additionally, we developed a customized library preparation protocol for ONT, significantly reducing the steps and time required for sample preparation. We also designed a bioinformatics pipeline for downstream analysis of sequencing reads for 16S rRNA profiling. Furthermore, we used both short (V3–V4) and long (V1–V9) reads to determine whether sequencing the full 16S rRNA gene provides new insights or deeper taxonomic classification compared to traditional short-read sequencing.

## Methods

### Animals

To generate *cxcl8a* deletion mutant in zebrafish, CRISPR-Cas9-mediated gene editing was performed using a guide RNA targeting the first exon of zebrafish *cxcl8a* (ENSDARG00000104795; GRCz11) sgRNA: the single guide used was AGGAATGAGCTTGAGAGGTC. The full sequence of the oligo used was [ccgctagc-taatacgactcactata-AGGAATGAGCTTGAGAGGTC–GTTTTAGAGCTAGAAATAGCAAG] and common REV primer [AAAAGCACCGACTCGGTGCCACTTTTTCAAGTTGATAACGGACTAGCCTTATTTTAA CTTGCTATTTCTAGCTCTAAAAC]. One-cell stage embryos were injected with 150 ng µl^−1^ sgRNA, 2 ng µl^−1^ Cas9 enzyme (IDT AltR s.p. Cas9 Nuclease V3 #1081058) and 0.3 µl phenol red. Analysis of the offspring of these F0 yielded heterozygous fish with different mutations in the germline. *cxcl8a* +/− F1 offspring (*n*=5 with different mutations) were selected and outcrossed to AB WT fish to yield F2 *cxcl8a* +/− zebrafish. The founder and offspring with a germline transmission of a 141 bp mutation in *cxcl8a* leading to a premature stop were selected and outcrossed to the double immune cell reporter transgenic Tg (mpeg1:mCherry/mpx:eGFPi^114^) to obtain *cxcl8a* +/+ Tg (mpeg1:mCherry/mpx:eGFPi^114^) and *cxcl8a* −/− (mpeg1:mCherry/mpx:eGFPi^114^) zebrafish harbouring GFP+ neutrophils and mCherry+ macrophages.

Adult *cxcl8a* +/+ Tg (mpeg1:mCherry/mpx:eGFPi^114^) and *cxcl8a* −/− (mpeg1:mCherry/mpx:eGFPi^114^) zebrafish were housed and fed as previously described [23]. Embryos were obtained by natural spawning and raised with E2 media (0.10 mM NaCl in demineralized water, pH 7.3) in Petri dishes at 28 °C (12/12 h light/dark cycle) [24]. Embryos were visually inspected daily, and visibly infected embryos were removed using an MS-222 (tricaine mesylate: tricaine/E3 (8.4% v/v 24 mM tricaine) (Sigma-Aldrich, DL, USA) solution.

### Experimental design

Fish were raised as previously described in [25] until 21 days post-fertilization (dpf). WT *cxcl8a* +/+ and mutant *cxcl8a* −/− fish were raised in separate tanks in triplicate. Fish were randomly sampled from each of three tanks.

### DNA extraction

Guts were extracted from juvenile zebrafish, rinsed in sterile PBS, snap-frozen in liquid nitrogen and preserved at −80 °C for total gut DNA extraction. DNA extractions were performed as previously described for each single gut [25, 26]. In brief, 100 µl lysis buffer (100 mM NaCl, 10 mM Tris pH 8, 15 mM EDTA 0.5% SDS) and 7 µl proteinase K (QIAGEN, 19131) were added to the samples. Samples were incubated at 56 °C overnight until fully dissolved. Then, 35 µl of 6M NaCl was added, and samples were incubated under continuous shaking. After centrifugation, the supernatant was transferred to a sterile 1.5 ml Eppendorf tube. Next, 270 µl of 100% (cold) ethanol was added, and samples were kept at −80 °C for 1 h. Samples were centrifuged for 5 min at full speed, and the pellet was washed with 70% ethanol. After centrifugation, the supernatant was removed, and pellets were air-dried before adding 20 µl RNase/DNase-free water. DNA concentrations were measured by Nanodrop and Qubit 2.0 fluorometer using Qubit^™^ dsDNA Quantification Assay Kits (Thermo Fisher Scientific, Q32853). The same samples were used for Illumina and Nanopore sequencing.

### Illumina sequencing

Next-generation sequencing using Illumina NovaSeq 6000 technology with the following forward (FWD) and reverse (REV) primers yielded 250 bp paired-end reads of the V3–V4 hypervariable region of the 16S rRNA gene: FWD (CCTACGGGNGGCWGCAG) and REV (GACTACHVGGGTATCTAATCC).

### 16S rRNA amplification and barcoding

PCR amplification reactions were performed using LongAmp polymerase (NEB, M0323S) according to the manufacturer’s instructions, with 100 ng of template DNA and a final reaction volume of 50 µl. Primers targeting the V3–V4 region of the 16S rRNA gene were provided by Baseclear B.V., allowing us to perform PCR amplification in our own lab using the same primers as those used by the company (Fig. 1). For the V1–V9 targeting region, we used previously published primers from Matsuo *et al.* [27] (Fig. 1, Table S1, available in the online Supplementary Material). Unique barcode sequences were added to the 5′ end of the FWD primers to ensure accurate sample identification during downstream data processing. The barcode sequences were extracted from the protocol of the Native Barcoding Kit 24 (ONT, SQK-NBD112.24) from Nanopore technologies. To obtain V3–V4 amplicons, thermal cycling conditions were as follows: 30 cycles, annealing temperature of 54 °C and extension time of 1 min. To obtain V1–V9 amplicons, the protocol was adjusted to an annealing temperature of 53 °C and an extension time of 2 min. In both protocols, 5 µl of PCR product was loaded onto a 0.8% agarose gel to confirm correct fragment amplification (data not shown). The remaining 45 µl of PCR product was purified using the Invisorb Fragment Clean-Up kit (Invitek Molecular, 1020300300) following the manufacturer’s instructions. Final DNA concentrations were measured using Qubit 2.0 fluorometer and Qubit^™^ dsDNA Quantification Assay Kits (Thermo Fisher Scientific, Q32853).

**Fig. 1.**
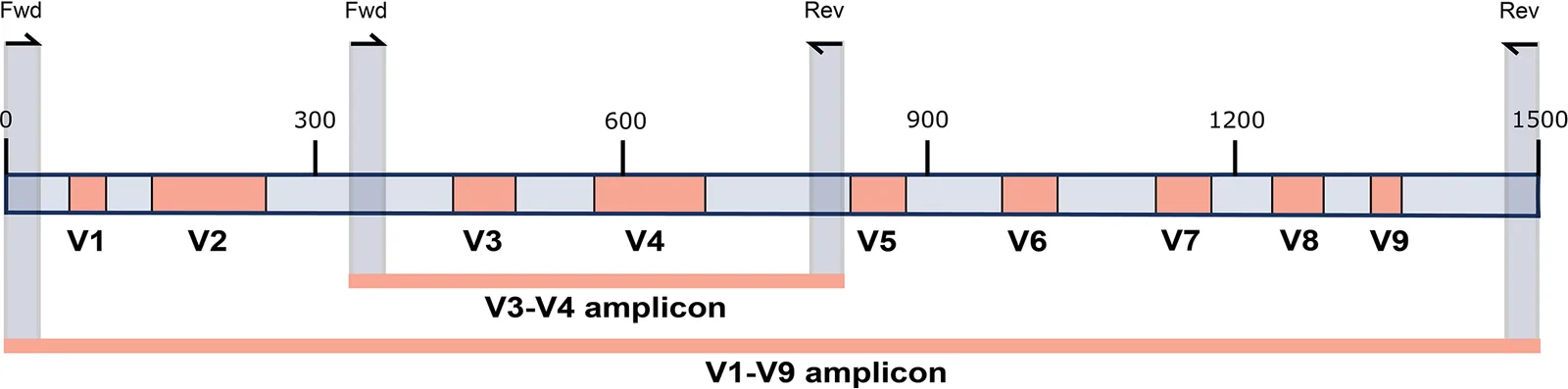
Schematic representation of the full-length 16S rRNA gene, highlighting conserved (grey) and variable (orange) regions (V1–V9). This visualization illustrates the structure of the gene and the relative coverage of the two amplicon strategies used in this study. The approximate locations of primer pairs targeting the V3–V4 and V1–V9 regions are indicated. Numbers along the gene correspond to nucleotide positions (in base pairs). Positions of each variable region were abstracted from study [37].

### Library preparation and sequencing

Sequencing was organized into batches of around 15 samples, each identified by barcodes ranging from 1 to 15 (see Table S1). Each batch of samples was pooled together in equimolar concentrations to a final concentration of 200 fmol. The library preparation was conducted using the Ligation Sequencing Kit V14 (ONT, SQK-LSK114) following the manufacturer’s instructions. Subsequently, the prepared library was loaded onto R10.4.1 flow cells (ONT, FLO-MIN114) using either the MinION Mk1c and Mk1b (ONT, Oxford, UK) sequencing devices, until most of the reads were sequenced. The total number of sequenced reads typically ranges from 14 to 19 million per flow cell, with the sequencing process usually taking between 24 and 48 h to complete, depending on the amplicon length.

### Sequencing data processing and downstream analysis

Raw Nanopore sequencing output was collected in .pod5 files and subsequently subjected to basecalling; results were stored in .fastq files using Dorado 0.5.3. Reads were demultiplexed using a custom script, available at [1].

The demultiplexed V3–V4 amplicon reads were then filtered by amplicon size: to obtain reads with sizes corresponding to the V3–V4 rRNA gene region, reads were filtered to 350–550 bp, while to obtain reads with sizes corresponding to the V1–V9 region, reads were filtered to 1,300–1,600 bp. Next, we classified reads based on their barcode sequences, retaining only those reads containing DNA sequences that matched our designed barcodes at 100% identity. This process resulted in 15 separate .fastq files, each .fastq file containing reads with a unique barcode, and an additional file for reads with no barcode or reads with less than 100% similarity (mismatches) to our barcodes. All reads passing these filtering steps were then processed using hidden Markov models to discard reads that did not resemble 16S rRNA. Finally, reads were trimmed to remove any sequences outside the primer regions, including the primer sequences themselves, retaining only the 16S rRNA fragments bordered by the respective primers. After this step, filtered and trimmed reads were parsed to the QIIME pipeline using default parameter settings for ASV calling with DADA2. For the V1–V9 amplicon reads, the ONT-AmpSeq workflow for OTU calling was used with the default parameters as recommended by the authors [2], followed by taxonomic classification and alpha- and beta-diversity analyses.

### Statistical analyses

Rarefaction curves are shown in Fig. S3. The Illumina and ONT V3–V4 datasets were rarefied to 50,000 reads per sample to enable direct comparison between sequencing platforms while retaining a comprehensive representation of taxonomic diversity. In contrast, the ONT V1–V9 dataset was analysed using all available reads, as rarefaction curves indicated that OTU richness continued to increase across the observed sequencing depths. Redundancy analysis (RDA) and principal component analysis (PCA) were performed in Canoco v5.15 [28] using analysis type ‘constrained’ or ‘unconstrained’, respectively. Response variables were log-transformed with the formula log(10,000*relative_abundance+1). RDA *P*-values were determined through permutation testing (*n*=500 permutations). Boxplots were generated using Prism v.9.0.0 (GraphPad Software, San Diego, CA, USA). Genus-level stacked bar plots were generated in R (v4.4.2) using relative abundance data. For each sequencing dataset, taxa were aggregated at the genus level, and mean relative abundances were calculated separately for WT and *cxcl8a* deletion mutant fish. Differential abundance analysis was performed at the genus level using ANCOM-BC2 in R (v4.4.2). For each sequencing dataset, ASV or OTU count tables were aggregated at the genus level and matched to sample metadata indicating genotype. Illumina V3–V4, ONT V3–V4 and ONT V1–V9 datasets were analysed independently using genotype as the explanatory variable. *P*-values were adjusted for multiple testing using the Benjamini–Hochberg false discovery rate procedure, and genera with a q-value below 0.05 were considered significantly differentially abundant. Log fold changes, standard errors, test statistics, *P*-values and q-values were exported for each dataset (Table S5). Results were visualized as volcano plots and summarized across sequencing approaches using a heatmap displaying significant genus-level log fold changes. Additional data handling and format conversions were done in Python 3.8.8 (https://www.python.org/).

## Results

### 16S rRNA sequencing workflow overview

We set out to design and validate a workflow to efficiently amplify and sequence a specific region of the 16S rRNA gene using ONT (see Methods). This workflow (Fig. 2) allows users to customize several parameters, including primer selection, amplicon size, barcode sequences and the number of samples to pool together. Additionally, PCR conditions, such as annealing temperature and extension times, can be adjusted based on user-defined criteria.

**Fig. 2.**
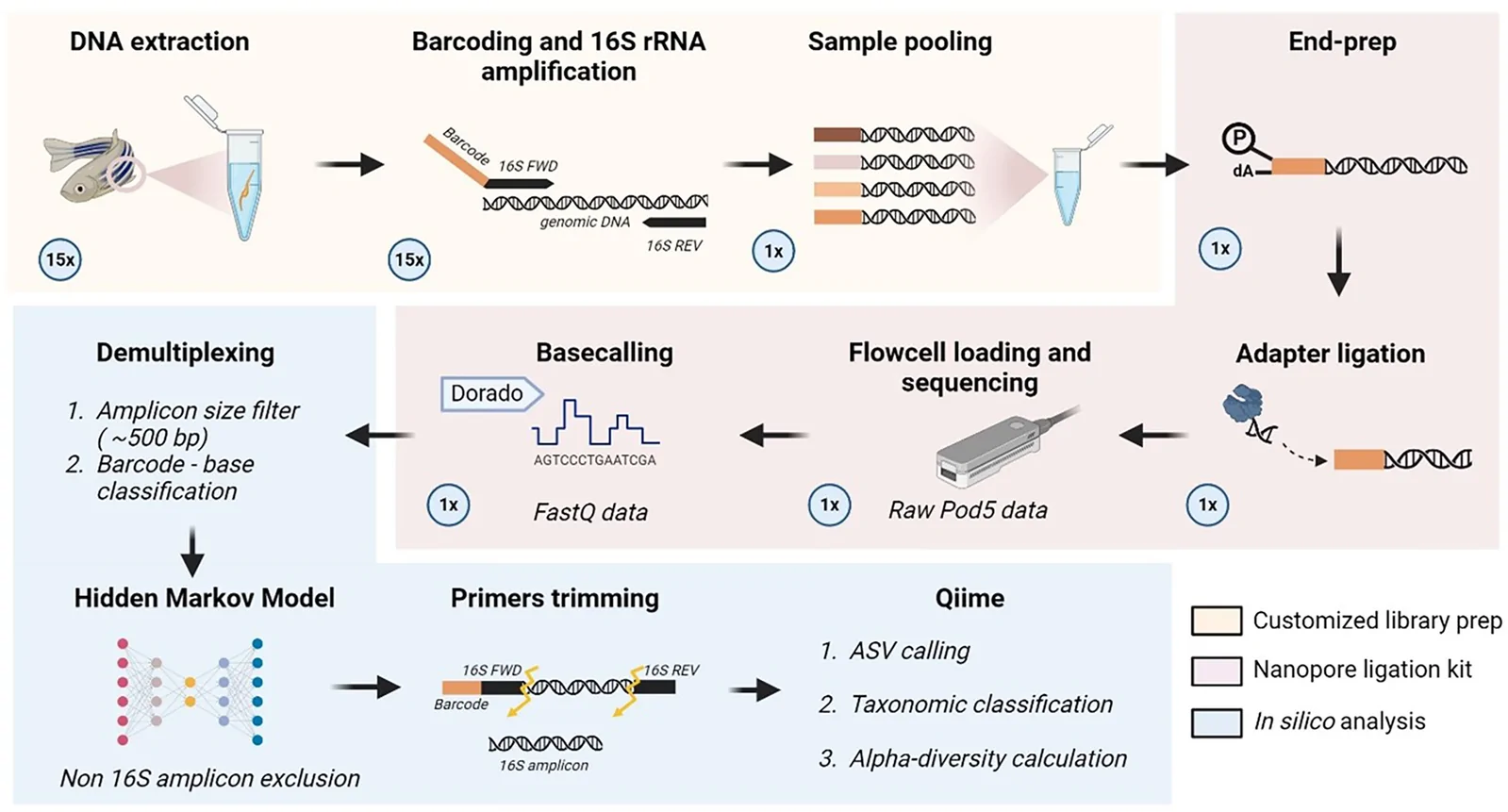
Overview of the customized ONT 16S rRNA sequencing workflow developed in this study. The workflow consists of three stages: (**i**) customized PCR-based library preparation incorporating sample-specific barcode overhangs, (ii) standard ONT ligation-based library preparation and sequencing and (iii) bioinformatic processing, including demultiplexing, quality filtering, trimming and downstream taxonomic analysis using either QIIME2 (V3–V4) or ONT-AmpSeq (V1–V9).

The workflow is divided into three main sections (Fig. 2). The first section involves a customized library preparation protocol adapted to the experimental design. This preparatory step was performed prior to initiating the ONT library preparation protocol. The second section includes steps using the Nanopore sequencing kit in accordance with the manufacturer’s instructions. The third and final section involves the bioinformatic analysis of the raw data, processed to facilitate ASV calling and taxonomic classification.

The choice of DNA extraction kit is independent of the subsequent workflow steps. For PCR amplification, users can select FWD and REV primers targeting the variable regions for the 16S rRNA gene. For this study, we performed PCR amplification targeting the V3–V4 and V1–V9 regions (Fig. 1). Each FWD primer included a barcode sequence as a single DNA oligo overhang at the 5′ end. Consequently, a unique DNA sequence, including a standard FWD primer, was used for each sample. Up to this point, each sample had been processed independently. Once samples had been tagged with a unique barcode, equimolar concentrations of each sample PCR amplicon were pooled together. This pooling step, enabled by our optimized preparation strategy, produced a single library adjusted to the final concentration required for the Nanopore ligation kit. For our study, we first performed a small preliminary trial to verify the workflow. As the sequencing output after bioinformatic processing of the complete workflow was still unknown, we conservatively decided to pool 15 samples per sequencing run to ensure sufficient read coverage during the initial validation of the method.

From this step onward, the protocol provided with the Nanopore ligation kit was implemented (Fig. 2), which consists of two main steps: (i) amplicon end-preparation and (ii) adapter ligation. After the adapter ligation step, the resulting DNA fragments were loaded onto the flow cell and sequenced until all PCR amplicons had been processed. Raw sequencing data were converted to .fastq files for downstream analysis.

### Data output comparison: Nanopore vs. Illumina

Due to the limitation of 15 barcodes and a total of 38 samples, sequencing was carried out in 3 rounds to accommodate all samples. Each round was performed separately for both the V3–V4 and V1–V9 amplicon regions, resulting in a total of six sequencing runs. Across all sequencing runs, 36.2 million reads were generated for the V3–V4 amplicons and 40.0 million reads for the V1–V9 amplicons before demultiplexing (Table S2).

To evaluate whether an excessive number of reads were discarded during any of the bioinformatic filtering steps and to assess the specificity of PCR, as well as the efficiency of the barcoding system (i.e. ensuring that no barcode interfered with 16S rRNA amplification due to non-specific binding elsewhere in the genome), we obtained detailed reports of reads per sample and total reads throughout the processing pipeline (Fig. 3, Table S2). In addition, unmatched reads were re-analysed using NanoSplit16S with an HMM-based 16S classifier. This analysis showed that 39.4% of unmatched V3–V4 reads and 27.3% of unmatched V1–V9 reads were still identified as 16S sequences (Table S4). These results indicate that the unmatched read fraction is heterogeneous and should not be interpreted exclusively as barcode assignment failures, as unsuccessful amplicon assignment may also result from other factors affecting barcode or primer recognition or the expected amplicon structure.

From these reports, we found that the read count distribution was more consistent in the V3–V4 samples, whereas the V1–V9 dataset exhibited greater variability, with outliers (Fig. 3) suggesting a less uniform equimolar input of the barcoded samples in V1–V9 compared to V3–V4.

**Fig. 3.**
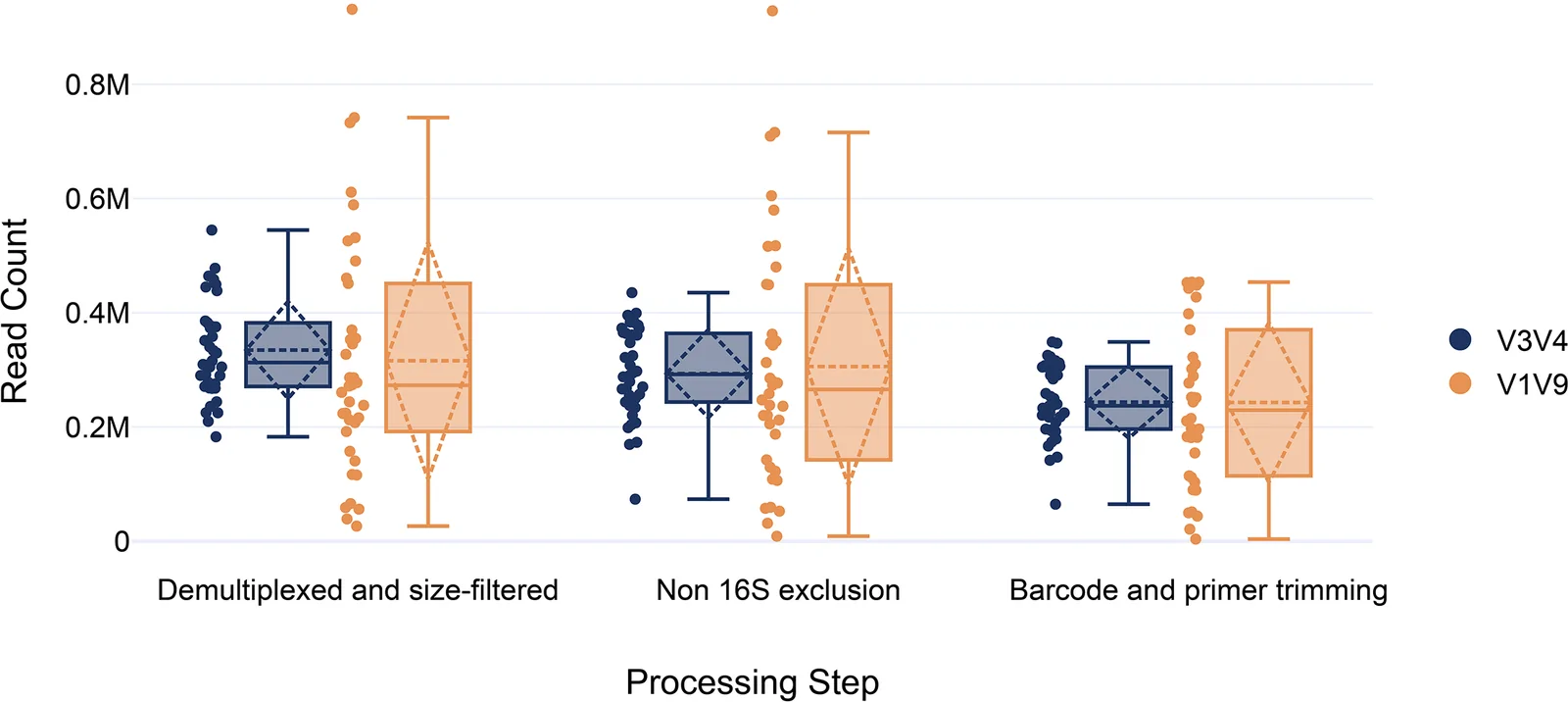
Distribution of read counts per sample across key bioinformatic processing steps for V3–V4 and V1–V9 16S rRNA gene amplicon datasets. Box plots depict the number of reads retained at each of three sequential steps. For each step, the blue and orange boxes represent the V3–V4 and V1–V9 datasets, respectively. Boxes display the interquartile range (IQR), with the median indicated by a horizontal line within the box. Individual sample values are plotted as points to illustrate variability.

The removal of non-16S rRNA amplicons (i.e. amplicons, including zebrafish DNA and DNA from other micro-organisms) had minimal impact on the total read count in both datasets, with 87.68 and 96.82% of average reads per sample retained for the V3–V4 and V1–V9 primer sets, respectively. Subsequent barcode and primer trimming steps further refined the datasets, retaining an average of 72.79% of reads per sample in the V3–V4 dataset and 76.88% in the V1–V9 dataset. A total average of 243,716 reads per sample in V3–V4 and 243,047 reads per sample in V1–V9 were retained for ASV or OTU assignment and taxonomic classification (Fig. 3, Table S2). Illumina sequencing yielded an average of 6.2 million reads per sample, provided directly by the sequencing company (Table S2).

### Alpha-diversity analysis of gut microbiome in mutant and WT zebrafish using V3–V4 amplicon sequencing by Illumina and Nanopore sequencing platforms

Gut samples (*n*=19/group) from *cxcl8a* +/+ and *cxcl8a* −/− fish (21 dpf) were used to determine prokaryotic community composition. All samples were sequenced using both amplicon sequencing of the V3–V4 16S rRNA gene region using Illumina (white background) and ONT (grey background) sequencing platforms (Fig. 4). The number of observed ASVs indexes (richness indicator) was comparable between *cxcl8a* +/+ and *cxcl8a* −/− zebrafish; Illumina sequencing identified a slight but significantly higher number of ASVs than ONT (Fig. 4a). Nonetheless, the increased number of ASVs did not reflect an increase in phylogenetic diversity (Fig. 4b). Other alpha-diversity metrics such as evenness or Shannon (diversity indicators) were comparable between genotypes and sequencing platforms (Fig. 4c, d).

**Fig. 4.**
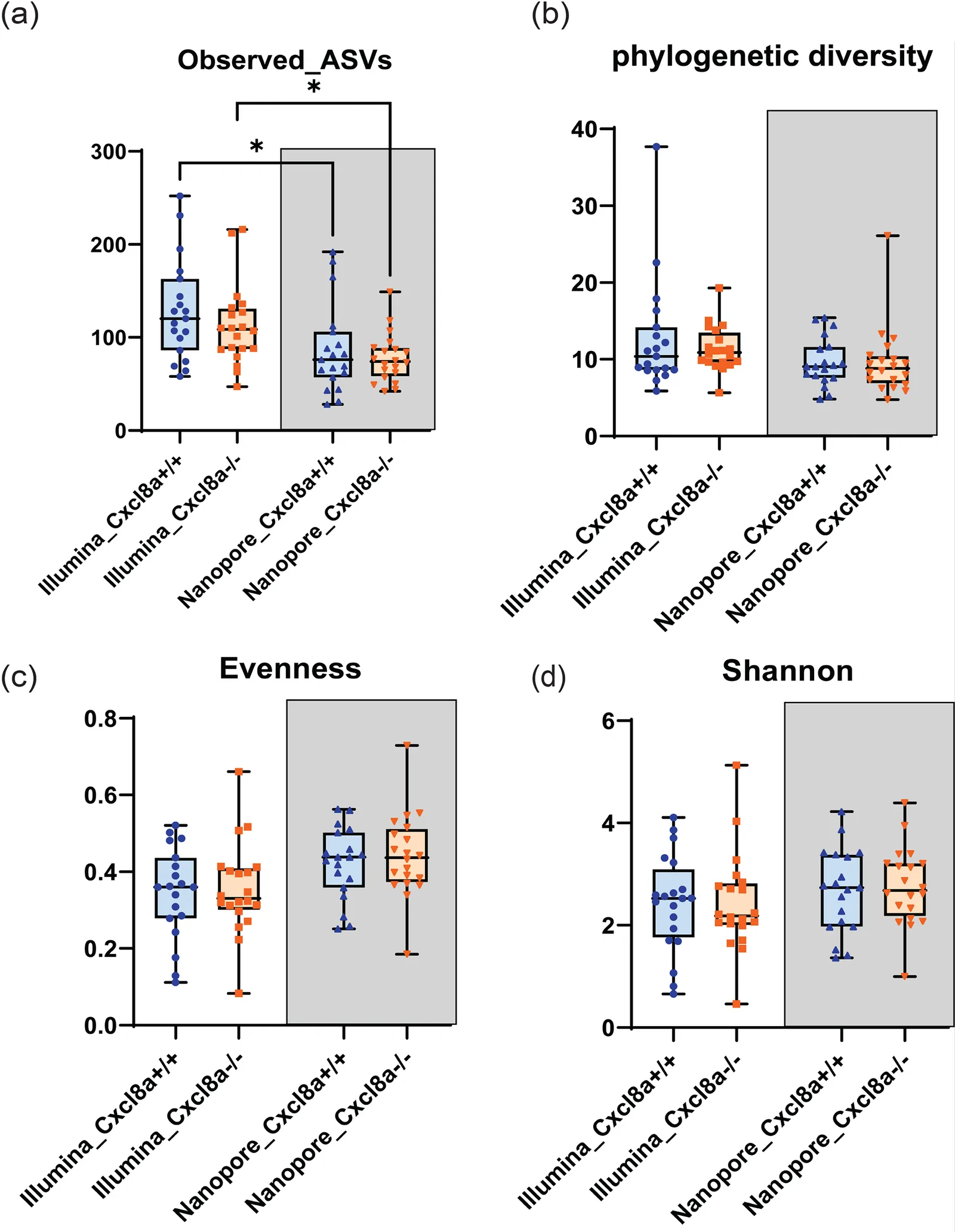
Alpha-diversity metrics of *cxcl8a +/+* and *cxcl8a −/−* zebrafish gut samples sequenced using Illumina (white background) and Nanopore (grey background) sequencing platforms. (a) Observed ASVs. (b) Phylogenetic diversity. (c) Evenness. (d) Shannon diversity index.

### Comparable beta-diversity in zebrafish gut microbiomes across sequencing platforms

To visualize the differences in the taxonomy and diversity of microbiota composition of the zebrafish gut resulting from Illumina or ONT platforms, we compared beta-diversity by PCA and RDA. Illumina NovaSeq 6000 and ONT sequencing explained roughly the same amount of variation in microbiota composition on the first two PCA axes, with PC1 explaining 27.88 and 25.57% of microbiota composition variation, respectively, and PC2 explaining 16.8% vs 17.62% of variation in microbiota composition, respectively (Fig. 5a). In the results of both sequencing platforms, samples similarly clustered in two separate groups depending on their genotype (*cxcl8a* +/+ and *cxcl8a* −/−). When comparing the variation in taxonomic composition of microbiota measured by Illumina and ONT sequencing platforms by RDA (*P*=0.002), the same trends were found as uncovered by PCA. PCA and RDA analyses identified the same genera as the most substantially different in relative abundance between WT and *cxcl8a* deletion mutant fish, as measured by both Illumina and ONT sequencing platforms.

**Fig. 5.**
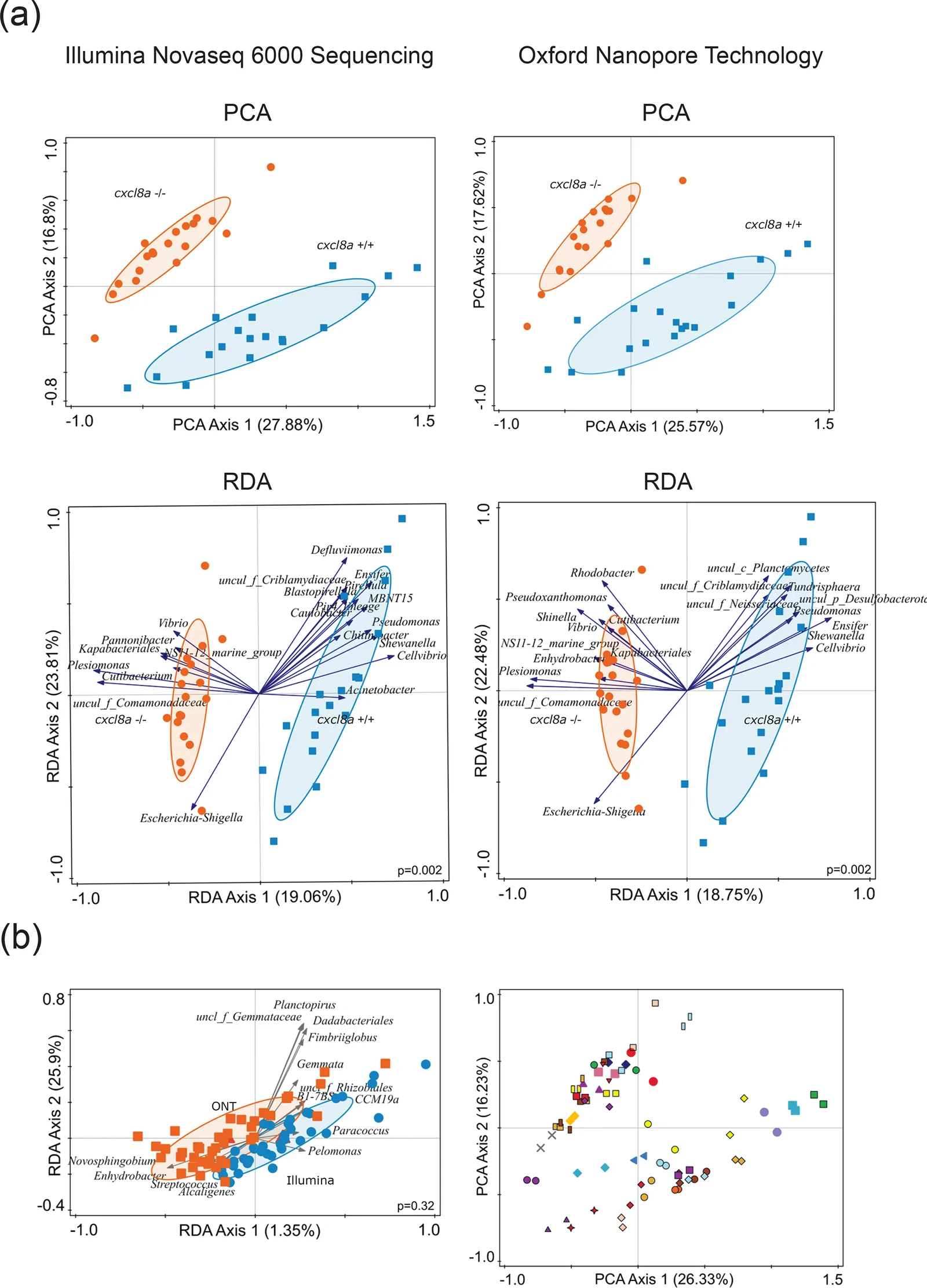
(a) Beta-diversity PCA and RDA of *cxcl8a* +/+ and *cxcl8a* −/− zebrafish gut samples. (b) Beta-diversity PCA and RDA of the two different sequencing technologies used in our study (Illumina and Nanopore).

To further explore whether the Illumina and ONT sequencing platforms capture different patterns in microbiota composition, we performed PCA and RDA analyses across all WT and *cxcl8a* deletion mutant samples (Fig. 5b). In contrast to platform-specific ordination, the total variation explained by PC1 and PC2 was comparable to the platform-specific analyses, suggesting overall consistency between sequencing methods. To visualize overlap between the two platforms at the sample level, we marked each sample with the same symbol across both platforms. Samples sequenced by both Illumina and ONT consistently clustered closely together, indicating that the microbiota profiles generated by each platform were highly similar.

Additionally, when all samples were included in a combined RDA, sequencing platform did not significantly explain the variation in microbial composition (*P*=0.32), further supporting the agreement between platforms across genotypes.

To further compare the taxonomic profiles generated by each sequencing approach, we visualized the mean relative abundance of the most abundant genera in WT and *cxcl8a* deletion mutant fish (Fig. 6). Illumina and ONT sequencing of the V3–V4 region produced highly similar community profiles, with the same dominant genera detected at comparable relative abundances in both genotypes. Both approaches identified a decrease in the relative abundance of *Pseudomonas* and *Aeromonas* and an increase in *Escherichia-Shigella* in the *cxcl8a* deletion mutant fish compared to WT fish.

**Fig. 6.**
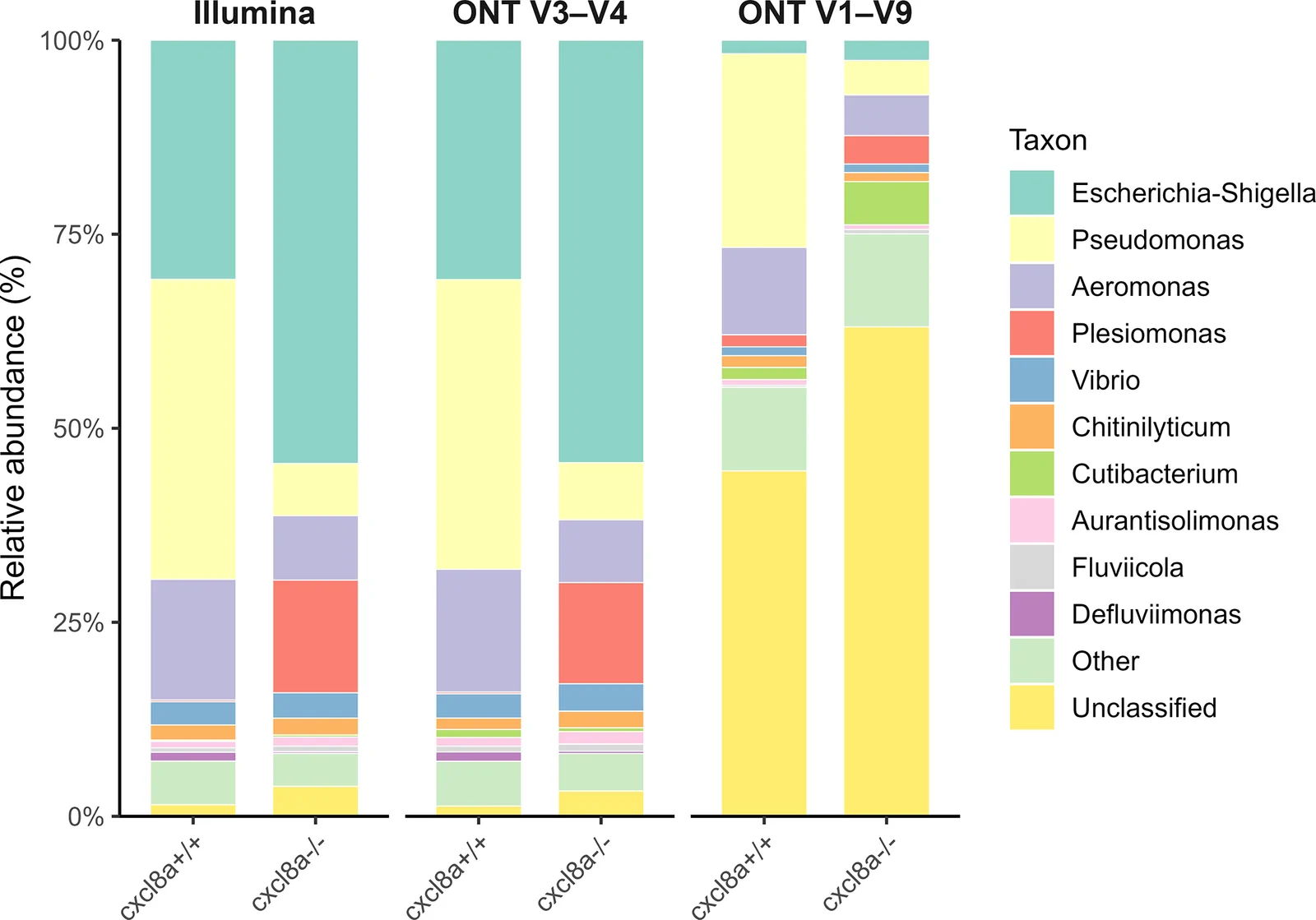
Relative abundance of dominant bacterial genera in WT and *cxcl8a* deletion mutant zebrafish across sequencing platforms. The ten most abundant classified genera across all samples and sequencing approaches were retained for visualization, while the remaining classified genera were grouped as ‘Other’. Taxa that could not be assigned at the genus level were grouped as ‘Unclassified’. Chloroplast-derived sequences were excluded from the visualization.

Finally, we wanted to assess whether genotype-associated differences in genus abundance were detected similarly by both sequencing techniques. To identify genera that differed between genotypes, we performed differential abundance analysis independently for the Illumina and ONT datasets (Figs 7a and S1). Based on these results, we selected a subset of genera and examined their relative abundances across individual samples (Fig. 7b).

**Fig. 7.**
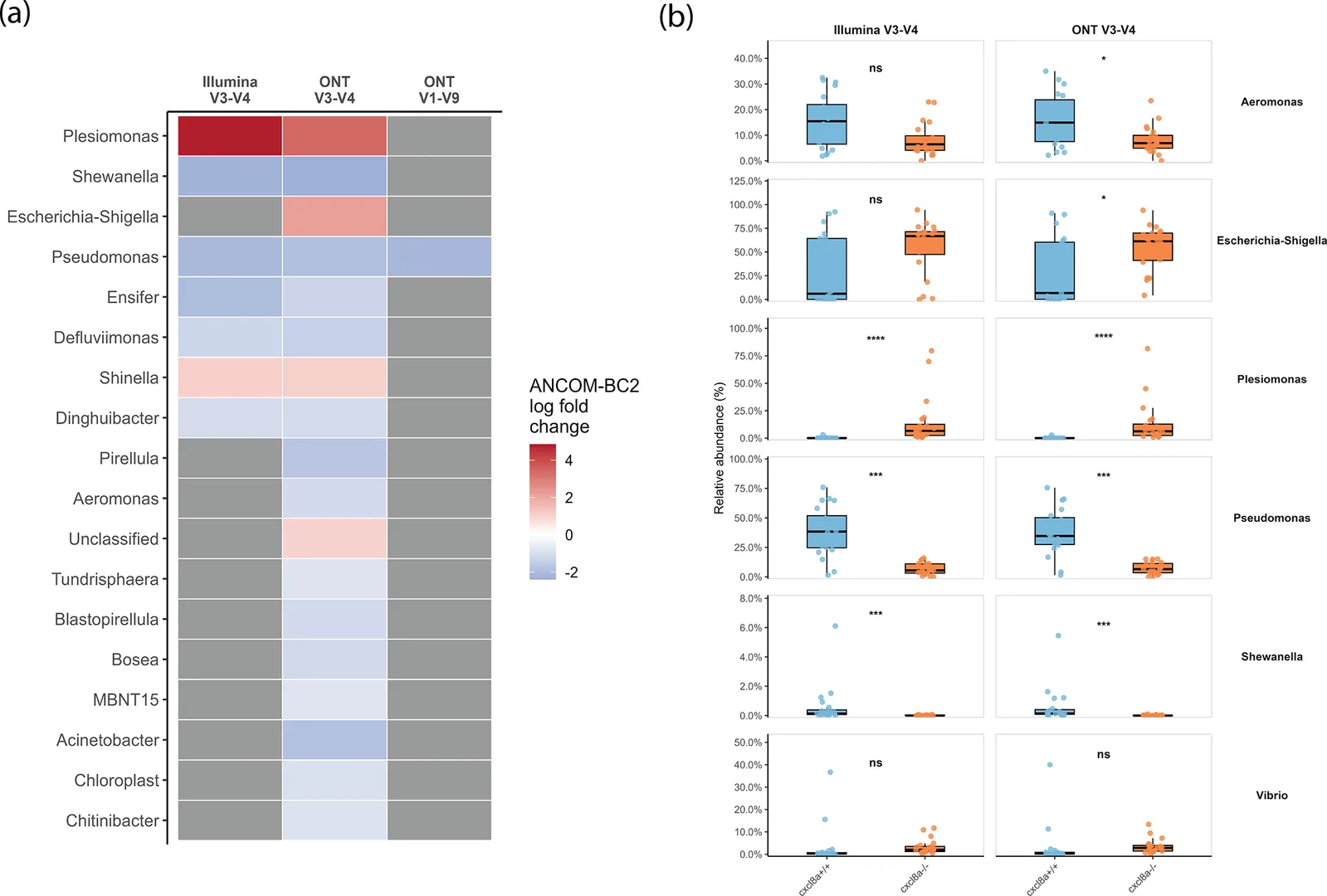
Differential abundance analysis of the zebrafish gut microbiota across sequencing platforms. (**a**) Heatmap showing the genera identified as significantly differentially abundant between WT and *cxcl8a* deletion mutant zebrafish in the Illumina V3–V4, ONT V3–V4 and ONT V1–V9 datasets. Colours indicate the ANCOM-BC2 log fold change (LFC), with red indicating higher abundance in mutant fish and blue indicating higher abundance in WT fish. Grey indicates that no significant difference was detected (q≥0.05). (**b**) Relative abundances of six selected genera across individual WT (*cxcl8a* +/+) and mutant (*cxcl8a* −/−) zebrafish sequenced using Illumina V3–V4 and ONT V3–V4. Boxes represent the IQR, the centre line indicates the median, whiskers extend to 1.5×IQR, and points represent individual samples. Statistical significance corresponds to the ANCOM-BC2 analysis: ns, q≥0.05; *q<0.05; **q<0.01; ***q<0.001; ****q<0.0001.

The selected genera included those identified as significantly differentially abundant between genotypes in both sequencing approaches (*Plesiomonas*, *Pseudomonas* and *Shewanella*). In addition, *Escherichia-Shigella* and *Aeromonas* were included because, although they reached statistical significance only in the ONT dataset, both genera displayed similar genotype-associated abundance trends (Fig. 6). Finally, *Vibrio* was included due to its previously reported biological relevance in zebrafish immune mutant models.

For the six genera analysed, both sequencing platforms identified highly similar abundance patterns between WT and mutant fish (Fig. 7b). Although *Aeromonas* and *Escherichia-Shigella* reached statistical significance after multiple testing correction only in the ONT dataset, both genera displayed similar abundance patterns and comparable log fold changes across sequencing platforms (Table S5). No significant differences between genotypes were detected for *Vibrio* in either sequencing approach.

### Evaluation of taxonomic diversity and resolution by 16S rRNA region and sequencing platform

Although Illumina and ONT generated highly comparable taxonomic profiles for the V3–V4 region (Fig. 6), taxonomic classification using the full-length V1–V9 amplicons produced markedly different genus-level profiles. Approximately 50% of reads remained unclassified at the genus level, substantially reducing the proportion of classified reads compared with the V3–V4 datasets (Fig. 6). Consequently, ANCOM-BC2 identified fewer significantly differentially abundant genera in the V1–V9 dataset than in either of the V3–V4 datasets (Fig. 7a). This reduction is likely attributable, at least in part, to the high proportion of unclassified reads, which limits the statistical power to detect genotype-associated differences at the genus level.

Following taxonomic classification using OTUs for V1–V9 and ASVs for V3–V4, we observed that the number of ASVs detected at each taxonomic level, from phylum to species, was consistent across Illumina and Nanopore sequencing platforms, suggesting that both platforms identify largely overlapping ASVs within the V3–V4 region (Fig. 8).

**Fig. 8.**
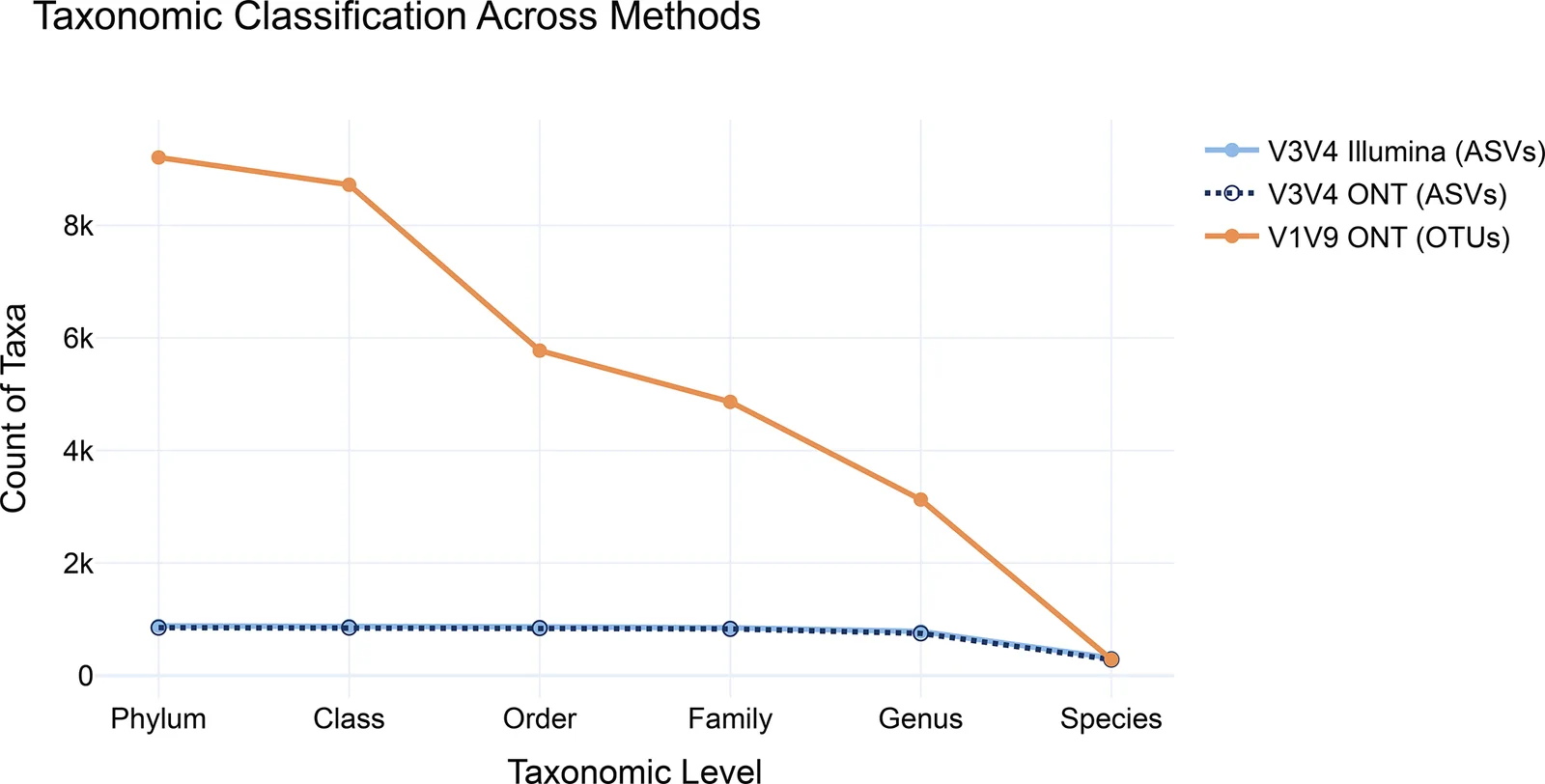
Taxonomic diversity of microbiota composition of zebrafish gut samples as measured by Illumina and ONT sequencing platforms. The number of identified taxa is shown at different taxonomic levels for V3–V4 (ASVs) using Illumina and ONT and V1–V9 (OTUs) using ONT.

In contrast, using the 16S rRNA V1–V9 region for taxonomic classification resulted in a substantial increase in the number of OTUs detected at each taxonomic level compared to the number of ASVs identified in the V3–V4 region, significantly improving taxonomic resolution (Fig. 8). Despite the higher taxonomic resolution observed with the full-length V1–V9 region, from phylum to genus, the number of taxa successfully assigned decreased with deeper classification levels. By the species level, the number of taxa assigned using V1–V9 was comparable to that obtained with the shorter V3–V4 region, although notable differences in specific taxa remained (Figs 8 and 9). Specifically, at the phylum level, taxonomic classification based on V3–V4 sequencing across all fish samples primarily identified two dominant taxa: Proteobacteria, which accounted for 94.3% of the classified sequences, and *Bacteroidota*, representing 2.5%. Taxonomic classification based on V1–V9 sequencing identified a broader range of taxa, including *Proteobacteria, Actinobacteriota, Bacteroidota, Firmicutes* and *Planctomycetota* (Fig. 9). At the genus level, there were notable differences in the relative abundances of taxa between the two amplicon regions (Fig. 9). For example, the genus *Cutibacterium* had a relative abundance of 3.6% in the V1–V9 analysis but was absent from the V3–V4 taxonomic assignment (Fig. 9). Additionally, a substantial difference was observed in the relative abundance of *Escherichia*-*Shigella*, which comprised 42% of the assigned taxa in the V3–V4 analysis, while only 2.2% of the taxa were assigned to this genus in the V1–V9 analysis (Fig. 9).

**Fig. 9.**
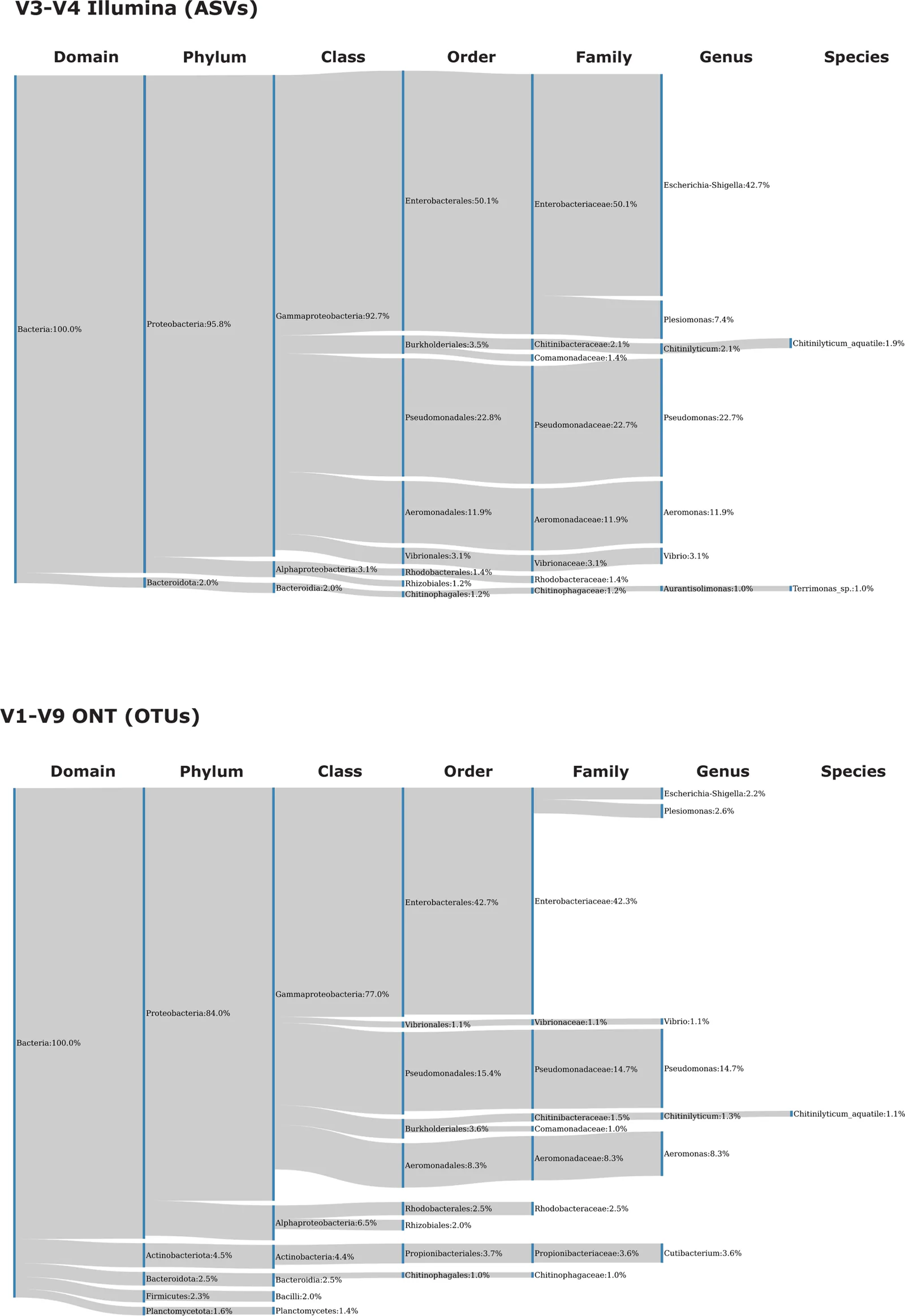
Sankey plots representing the taxonomy classification of microbial communities using (a) ASV-based classification from V3–V4 regions and (b) OTU-based classification from Nanopore sequencing of the V1–V9 region. Taxonomic abundances are based on pooled read counts across all samples for each sequencing method. Percentages represent the relative abundance of the aggregated dataset.

To further assess the influence of the taxonomic classification pipeline, the V1–V9 dataset was also analysed using the EPI2ME workflow (Fig. S2). Compared with the ONT-AmpSeq pipeline, EPI2ME assigned a greater proportion of reads to the species level but recovered two fewer phyla than the ONT-AmpSeq analysis.

### Nanopore sequencing demonstrates a lower per-sample cost compared to Illumina

Nanopore library preparation and sequencing costs were estimated from DNA extraction-ready samples through sequencing data generation. The workflow consisted of two main components: the customized library preparation and the ONT sequencing kit (Fig. 2). During library preparation, DNA samples were amplified using an FWD primer containing a 5′ barcode overhang, followed by PCR clean-up (Fig. 2; see Methods). In our workflow, the reagent cost of attaching barcodes to PCR amplicons was €1.84 per sample. Samples were pooled prior to the end-preparation and barcode ligation steps, which were performed in a single reaction. The total reagent cost of the workflow was €606.09 per sequencing run (Table S3). When 15 samples were multiplexed in a single run, this corresponded to an estimated reagent cost of ~€40.4 per sample. In comparison, the cost of outsourced Illumina NovaSeq sequencing was €156 per sample, representing an ~3.9-fold lower per-sample cost for the ONT workflow under the conditions evaluated in this study (Table S3).

## Discussion

In this study, we developed a custom PCR barcoding strategy optimized for ONT 16S rRNA sequencing. We then demonstrated that the taxonomic classifications obtained from the V3–V4 region were highly consistent with those generated by the current gold standard, Illumina NovaSeq. Previous studies utilizing ONT for 16S rRNA sequencing have faced challenges in ASV identification (i.e. taxonomic classification based on sequence variations at the single-nucleotide level) due to high error rates [29] and the reliance on ligation-based barcoding, which is costly, labour-intensive and prone to biases [30, 31]. Our approach was effective in a real biological case study of *cxcl8a +/+* and *cxcl8a −/−* zebrafish, enabling ASV-level taxonomic classification while reducing per-sample sequencing costs and processing time. Furthermore, we extended our analysis to compare full-length 16S rRNA V1–V9 sequencing with short-read V3–V4 sequencing, revealing an increased number of OTUs in long-read sequencing, though with some discrepancies in taxonomic assignments. This highlights the trade-offs between improved richness and potential classification challenges due to limitations in current 16S rRNA reference databases and the lack of optimized bioinformatics pipelines for full-length 16S rRNA gene analysis.

A major challenge in ONT-based taxonomic classification is the reliance on alignment-based bioinformatics pipelines, such as EPI2ME and EMU [32, 33]. These methods do not perform *de novo* clustering, limiting the identification of OTUs or ASVs, which are essential for high-resolution microbiome analysis [29, 33]. Our results demonstrate that ONT short-read sequencing can achieve ASV classification with an accuracy comparable to Illumina when processed with an optimized bioinformatics pipeline. Using the latest ONT chemistry (V14) and the Dorado basecalling model, we obtained taxonomic classifications nearly identical to those generated by the current gold standard Illumina NovaSeq sequencing platform, confirming that the ONT sequencing platform is capable of resolving complex microbial community composition at high resolution. Although Illumina sequencing detected more ASVs, these additional sequence variants did not translate into substantial differences in biological interpretation. Alpha- and beta-diversity metrics, genus-level community composition and differential abundance analyses showed a high degree of agreement between Illumina and ONT V3–V4 sequencing. While ONT identified a greater number of significantly differentially abundant genera after multiple testing correction, the dominant genotype-associated abundance patterns and estimated log fold changes were highly comparable between sequencing platforms. Together, these findings demonstrate that ONT short-read sequencing reliably captures biologically meaningful microbiome differences comparable to those obtained with Illumina sequencing.

Recent advancements, such as the ONT-AmpSeq pipeline, have improved taxonomic resolution by enabling OTU classification of full-length 16S rRNA sequences [2], raising interest in the potential benefits of long-read sequencing for microbiome profiling. To evaluate the taxonomic resolution of full-length 16S rRNA sequencing, we applied OTU-based classification via the ONT-AmpSeq workflow to ONT V1–V9 long sequencing reads and compared them to the ASV-based classification of V3–V4 reads from both ONT and Illumina.

While full-length 16S rRNA gene sequencing detected a greater number of OTUs, approximately half of the V1–V9 reads remained unclassified at the genus level. This reduced the amount of information available for genus-level analyses and likely contributed to the smaller number of significantly differentially abundant genera identified compared with the V3–V4 datasets [34, 35]. A marked discrepancy was observed in *Escherichia-Shigella* abundance (42.7% in V3–V4 versus 2.2% in V1–V9). This discrepancy likely reflects a combination of differences in the sequenced 16S rRNA region, reference database completeness and the taxonomic classification workflow used [34, 36]. Interestingly, analysis of the same V1–V9 dataset using the EPI2ME workflow produced a different taxonomic profile. Compared with the ONT-AmpSeq pipeline, EPI2ME identified fewer phyla but assigned a larger proportion of reads to the species level.

This reinforces previous findings that taxonomic resolution is highly dependent on the selected variable region, as different bacterial genera exhibit varying levels of conservation across the 16S rRNA gene [34, 35, 37] and also on the bioinformatic workflow used for taxonomic assignment. Full-length 16S rRNA sequencing has the potential to address discrepancies in microbiota composition that arise from the use of different variable regions across studies. However, improvements are still needed in reference databases containing full-length 16S rRNA genes, as well as in the algorithms used to distinguish true biological variation from sequencing errors [35].

From a microbial ecology point of view, gut microbial compositions found in WT zebrafish are comparable to previously found results. As reviewed in López Nadal *et al.* [38], the WT gut zebrafish microbiota is mostly hosting Gamma-Proteobacteria which includes the following genera: *Escherichia*, *Pseudomonas*, *Plesiomonas*, *Aeromonas*, *Shewanella* and *Vibrio*, among others, found in guts of WT fish in the present study. Interestingly, some of these genera are found significantly altered in *cxcl8a* deletion mutant (*Aeromonas*, *Escherichia-Shigella*, *Plesiomonas*, *Pseudomonas* and *Shewanella*) and with relevant trends (*Vibrio*) when compared to WT (Fig. 7). Current efforts are expanding on these data to further elucidate the impact of Cxcl8a on microbial composition in the zebrafish gut.

We developed a simplified and cost-effective PCR-based barcoding strategy that eliminates the need for ligation-based barcoding, thereby streamlining the workflow. Both standard ONT barcoding kits and more recent approaches aimed at improving barcoding efficiency rely on ligation-based workflows or additional amplification steps, which increase workload and may introduce bias [30, 31, 39–41]. Our approach directly incorporates barcodes during the initial PCR amplification step, enabling simultaneous preparation of multiple samples. In this study, up to 15 samples were pooled. The consistent average read counts per sample obtained from both V3–V4 and V1–V9 ONT sequencing indicate that our PCR-based barcoding strategy is effective regardless of amplicon length and the primers used. For the V3–V4 datasets, rarefaction analysis further confirmed that a sequencing depth of around 50,000 reads per sample is sufficient to capture the full taxonomic diversity, demonstrating that our approach not only yields sample sequencing depth but is also well-suited for studying complex microbial communities, such as those found in the zebrafish gut. Additionally, by allowing flexible primer selection, our approach overcomes the constraint of predefined 16S rRNA primer sets, offering greater adaptability for microbiome studies [30, 31].

In our study, we employed a conservative pooling strategy of 15 samples per flow cell and still achieved an ~3.9-fold lower per-sample sequencing cost than outsourced Illumina NovaSeq sequencing under the conditions evaluated in this study. Subsequent studies in our lab and in collaborating groups have applied the same workflow across diverse biological contexts, including 16S rRNA gut microbiome profiling of bees (data not published), zebrafish gut microbiota analyses [42] and vertebrate and plant metabarcoding of hyena scats collected across multiple sites in southern and eastern Africa [43]. In the latter, both vertebrate 12S rRNA and plant ITS2 markers were successfully targeted, extending the applicability of the workflow beyond bacterial communities. Notably, the hyena scat study was conducted directly in field laboratories. Across these applications, between 36 and up to 75 samples were multiplexed per flow cell, further reducing per-sample costs and demonstrating the scalability and portability of the workflow beyond the conditions tested here.

In conclusion, our study highlights the robustness of ONT short-read sequencing for microbiome profiling while maintaining comparable taxonomic classification accuracy as the Illumina sequencing platform. While full-length sequencing holds promise for capturing greater taxonomic diversity, classification accuracy remained constrained by database limitations and lack of optimized algorithms. By developing and implementing an optimized PCR-based barcoding strategy, we streamlined typical 16S rRNA gene sequencing workflows, reducing processing time and costs while enabling the simultaneous preparation of multiple samples. This adapted approach balances cost, accuracy and scalability and offers valuable insights into the future of nanopore-based microbiome research and taxonomic classifications.

## Supplementary material

10.1099/mgen.0.001828 Supplementary Material 1.

## Abbreviations

ASVs: amplicon sequence variants
dpf: days post-fertilization
FWD: forward
IQR: interquartile range
ONT: Oxford Nanopore Technologies
OTUs: operational taxonomic units
PCA: principal component analysis
RDA: redundancy analysis
REV: reverse
