# Supplementary material for: A scalable and cost-effective nanopore workflow for 16S rRNA microbiome profiling validated in a zebrafish gut model
Source: Microb Genom. 2026 Sep 29;12(9):001828. doi: 10.1099/mgen.0.001828 (PMC13623021; doi:10.1099/mgen.0.001828)

Table S1. Primer sequences used in this study, with barcode sequences in italics.

**Name**	**Sequence**
V3-V4 Bc1 FWD	*CACAAAGACACCGACAACTTTCTT*CCTACGGGNGGCWGCAG
V3-V4 Bc2 FWD	*ACAGACGACTACAAACGGAATCGA*CCTACGGGNGGCWGCAG
V3-V4 Bc3 FWD	*CCTGGTAACTGGGACACAAGACTC*CCTACGGGNGGCWGCAG
V3-V4 Bc4 FWD	*TAGGGAAACACGATAGAATCCGAA*CCTACGGGNGGCWGCAG
V3-V4 Bc5 FWD	*AAGGTTACACAAACCCTGGACAAG*CCTACGGGNGGCWGCAG
V3-V4 Bc6 FWD	*GACTACTTTCTGCCTTTGCGAGAA*CCTACGGGNGGCWGCAG
V3-V4 Bc7 FWD	*AAGGATTCATTCCCACGGTAACAC*CCTACGGGNGGCWGCAG
V3-V4 Bc8 FWD	*ACGTAACTTGGTTTGTTCCCTGAA*CCTACGGGNGGCWGCAG
V3-V4 Bc9 FWD	*AACCAAGACTCGCTGTGCCTAGTT*CCTACGGGNGGCWGCAG
V3-V4 Bc10 FWD	*GAGAGGACAAAGGTTTCAACGCTT*CCTACGGGNGGCWGCAG
V3-V4 Bc11 FWD	*TCCATTCCCTCCGATAGATGAAAC*CCTACGGGNGGCWGCAG
V3-V4 Bc12 FWD	*TCCGATTCTGCTTCTTTCTACCTG*CCTACGGGNGGCWGCAG
V3-V4 Bc13 FWD	*AGAACGACTTCCATACTCGTGTGA*CCTACGGGNGGCWGCAG
V3-V4 Bc14 FWD	*AACGAGTCTCTTGGGACCCATAGA*CCTACGGGNGGCWGCAG
V3-V4 Bc15 FWD	*AGGTCTACCTCGCTAACACCACTG*CCTACGGGNGGCWGCAG
V3-V4 REV	GACTACHVGGGTATCTAATCC
V1-V9 Bc1 FWD	*CACAAAGACACCGACAACTTTCTT*AGRGTTYGATYMTGGCTCAG
V1-V9 Bc2 FWD	*ACAGACGACTACAAACGGAATCGA*AGRGTTYGATYMTGGCTCAG
V1-V9 Bc3 FWD	*CCTGGTAACTGGGACACAAGACTC*AGRGTTYGATYMTGGCTCAG
V1-V9 Bc4 FWD	*TAGGGAAACACGATAGAATCCGAA*AGRGTTYGATYMTGGCTCAG
V1-V9 Bc5 FWD	*AAGGTTACACAAACCCTGGACAAG*AGRGTTYGATYMTGGCTCAG
V1-V9 Bc6 FWD	*GACTACTTTCTGCCTTTGCGAGAA*AGRGTTYGATYMTGGCTCAG
V1-V9 Bc7 FWD	*AAGGATTCATTCCCACGGTAACAC*AGRGTTYGATYMTGGCTCAG
V1-V9 Bc8 FWD	*ACGTAACTTGGTTTGTTCCCTGAA*AGRGTTYGATYMTGGCTCAG
V1-V9 Bc9 FWD	*AACCAAGACTCGCTGTGCCTAGTT*AGRGTTYGATYMTGGCTCAG
V1-V9 Bc10 FWD	*GAGAGGACAAAGGTTTCAACGCTT*AGRGTTYGATYMTGGCTCAG
V1-V9 Bc11 FWD	*TCCATTCCCTCCGATAGATGAAAC*AGRGTTYGATYMTGGCTCAG
V1-V9 Bc12 FWD	*TCCGATTCTGCTTCTTTCTACCTG*AGRGTTYGATYMTGGCTCAG
V1-V9 Bc13 FWD	*AGAACGACTTCCATACTCGTGTGA*AGRGTTYGATYMTGGCTCAG
V1-V9 Bc14 FWD	*AACGAGTCTCTTGGGACCCATAGA*AGRGTTYGATYMTGGCTCAG
V1-V9 Bc15 FWD	*AGGTCTACCTCGCTAACACCACTG*AGRGTTYGATYMTGGCTCAG
V1-V9 REV	CGGYTACCTTGTTACGACTT

Table S2		**Reads per flow cell**	**Total reads before demultiplexing***
**V3V4**	**Batch 1**	9441800	36158926
	**Batch 2**	15217607	
	**Batch 3**	11499519	
**V1V9**	**Batch 1**	15278074	40027526
	**Batch 2**	9379759	
	**Batch 3**	15369693	

**Total reads are reported per sequencing run before demultiplexing. Read counts for individual samples are reported only after barcode-based demultiplexing.*

**V3V4**				**V1V9**			**Illumina**
**Sample_name_tube**	Demultiplexed and size-filtered	Non 16S exclusion	Barcode and primer trimming	Demultiplexed and size-filtered	Non 16S exclusion	Barcode and primer trimming	
DNA_21dpf_--_1	316117	297772	237764	732970	709419	451077	6825935
DNA_21dpf_--_2	309734	279665	225119	58983	57748	51384	6154087
DNA_21dpf_--_3	182927	169574	147296	116017	106513	89363	7222003
DNA_21dpf_--_5	464405	395808	346794	116938	108614	89985	5156175
DNA_21dpf_--_6	385783	372419	300191	611254	605089	452926	7430708
DNA_21dpf_--_7	225050	198863	173774	223800	220510	185672	6321965
DNA_21dpf_--_8	438742	399434	325564	140668	129631	111844	6129337
DNA_21dpf_--_9	458552	383762	307679	157435	122592	103306	6461504
DNA_21dpf_--_10	275495	237778	191688	271800	247506	215114	6227121
DNA_21dpf_--_11	292152	207449	179229	192151	187513	154607	6950975
DNA_21dpf_--_12	236119	220725	192223	260716	258239	210677	6493893
DNA_21dpf_--_13	544921	382802	306573	741905	715909	450766	6961246
DNA_21dpf_--_14	375459	361283	290761	525982	517462	453795	7028298
DNA_21dpf_--_15	449911	435287	349016	931633	928856	448272	5989241

DNA_21dpf_--_16	478070	363982	290976	286674	278870	251256	6364353
DNA_21dpf_--_17	358594	287882	232984	589211	579926	453574	6392533
DNA_21dpf_--_18	445217	360945	312251	244223	238273	197348	3886928
DNA_21dpf_--_19	329745	325056	284235	238134	236563	196512	6088639
DNA_21dpf_--_20	267630	255500	208129	277795	277124	252170	5505566
DNA_21dpf_++_1	290347	73684	64827	65948	59491	49752	5814268
DNA_21dpf_++_2	289972	250015	217300	353198	348341	289420	6281921
DNA_21dpf_++_3	373611	373116	323195	451442	449115	370268	6494850
DNA_21dpf_++_4	305221	297864	236828	327363	312962	277131	5484736
DNA_21dpf_++_5	271100	243892	196451	370012	362692	322425	7570103
DNA_21dpf_++_6	225161	173177	141819	490852	480301	427681	6134729
DNA_21dpf_++_7	244214	234138	194940	460774	449967	398336	6099665
DNA_21dpf_++_8	278367	270299	224922	26524	8800	3769	6337121
DNA_21dpf_++_9	267977	257286	224740	39106	31575	21141	6585737
DNA_21dpf_++_11	309827	308087	249786	345515	343511	301911	5830582
DNA_21dpf_++_12	376435	347789	304988	56211	52678	44064	6362861
DNA_21dpf_++_13	333858	290069	253404	224459	220327	183127	5573025
DNA_21dpf_++_14	463814	365640	316337	216171	212232	181503	5598424
DNA_21dpf_++_15	209957	204098	166720	531699	516465	442917	6628330
DNA_21dpf_++_16	382515	378262	305108	274223	273348	243995	6303091
DNA_21dpf_++_17	268398	266647	217832	286372	285482	251855	6334095
DNA_21dpf_++_18	351482	321861	258796	207800	205578	181942	6084817
DNA_21dpf_++_19	340434	268187	220725	212671	142631	114524	5426807
DNA_21dpf_++_20	305190	294426	240248	355250	350400	310387	7583561
**avarage reads per sample**	334802,71	293540,08	243716,11	316154,71	306111,92	243047,26	6266295,53
**retained reads per step (%)**		87,68	83,03		96,82	79,40	

Table S3. Library preparation and sequencing costs for the ONT workflow and outsourced Illumina NovaSeq sequencing.. Cost estimates exclude consumables and working hours.

**ONT**							
	Product	Specific product	Price/unit (€)	Reactions/unit	Price/reaction (€)	Reactions	ONT (€)
Barcoding PCR	Polymerase	LongAmp Hot Start Taq	199	500	0,4	15	6
	PCR clean-up kit	InviSorb® Fragment CleanUp	359	250	1,44	15	21,6
Sequencing	ONT kit	Ligation sequencing kit V14 (SQK-LSK114)	570	6	95	1	95
	3rd party reagents	Ultra II End-prep enzyme mix	287	24	11,9	1	11,9
		Quick T4 DNA ligase	1459	100	14,59	1	14,59
	Flowcell	FLO-MIN114	475	1	475	1	457
Total price							606,09
Price per sample							40,4
**Illumina NovaSeq**							
Price per sample	156						

Table S4. HMM-based identification of 16S rRNA gene sequences among unmatched reads generated during the demultiplexing step of the bioinformatic workflow.

Amplicon	Total_unmatched_reads	HMM_16S_reads	HMM_unassigned_reads	Percent_16S_in_unmatched
**V3V4**	14940341	5881966	9058375	39.37
**V1V9**	15796169	4307582	11488587	27.27

Table S5. Comparison of ANCOM-BC2 differential abundance results across sequencing methods. Log fold changes (LFC), adjusted *q*-values, significance status (FDR < 0.05).

**genus**	**lfc_illumina**	**q_illumina**	**sig_illumina**	**lfc_ont_v3v4**	**q_ont_v3v4**	**sig_ont_v3v4**	**lfc_ont_v1v9**	**q_ont_v1v9**	**sig_ont_v1v9**	**n_methods_significant**
Pseudomonas	-2,097	0,001	TRUE	-1,920	0,000	TRUE	-2,157	0,012	TRUE	3
Defluviimonas	-1,220	0,012	TRUE	-1,368	0,001	TRUE				2
Dinghuibacter	-1,057	0,020	TRUE	-1,083	0,011	TRUE	0,469	0,298	FALSE	2
Ensifer	-2,022	0,000	TRUE	-1,286	0,001	TRUE				2
Plesiomonas	4,848	0,000	TRUE	3,418	0,000	TRUE	1,437	0,105	FALSE	2
Shewanella	-2,334	0,000	TRUE	-2,390	0,000	TRUE	0,183	0,838	FALSE	2
Shinella	1,082	0,039	TRUE	1,057	0,011	TRUE				2
Acinetobacter	-0,502	0,295	FALSE	-1,904	0,000	TRUE	-0,504	0,423	FALSE	1
Aeromonas	-0,907	0,096	FALSE	-1,090	0,031	TRUE	-0,557	0,197	FALSE	1
Blastopirellula	-0,818	0,084	FALSE	-1,090	0,007	TRUE	-0,655	0,222	FALSE	1
Bosea	-0,801	0,096	FALSE	-1,156	0,004	TRUE	-0,601	0,271	FALSE	1
Chloroplast	-0,317	0,527	FALSE	-0,937	0,046	TRUE	0,308	0,580	FALSE	1
Escherichia-Shigella	2,197	0,088	FALSE	2,200	0,046	TRUE	0,955	0,253	FALSE	1
MBNT15	-0,689	0,096	FALSE	-0,777	0,035	TRUE				1
Pirellula	-0,973	0,063	FALSE	-1,702	0,001	TRUE	-0,235	0,694	FALSE	1
Tundrisphaera	-0,864	0,090	FALSE	-0,852	0,040	TRUE	-0,323	0,542	FALSE	1
Unclassified	0,884	0,066	FALSE	1,016	0,048	TRUE	0,658	0,175	FALSE	1
Chitinibacter				-0,896	0,048	TRUE	0,421	0,326	FALSE	1
Allorhizobium-Neorhizobium-Pararhizobium-Rhizobium	-0,119	0,788	FALSE				-0,266	0,665	FALSE	0
Arenimonas	-0,570	0,295	FALSE	-0,472	0,361	FALSE				0
Aurantisolimonas	0,182	0,777	FALSE	-0,005	0,989	FALSE	-0,108	0,863	FALSE	0

Babeliaceae	-0,339	0,399	FALSE				-0,154	0,721	FALSE	0
Bdellovibrio	0,510	0,205	FALSE	0,014	0,971	FALSE	0,134	0,863	FALSE	0
Bradyrhizobium	-0,241	0,675	FALSE	-0,417	0,342	FALSE	-0,254	0,646	FALSE	0
CL500-3	-0,144	0,788	FALSE	-0,136	0,764	FALSE	-0,037	0,978	FALSE	0
Candidatus_Alysiosphaera	-0,277	0,520	FALSE	-0,414	0,243	FALSE	-0,068	0,863	FALSE	0
Chitinilyticum	-0,089	0,910	FALSE	-0,302	0,602	FALSE	-0,454	0,665	FALSE	0
Corynebacterium	-0,754	0,096	FALSE	0,358	0,484	FALSE	0,181	0,668	FALSE	0
Cutibacterium	1,042	0,096	FALSE	0,855	0,167	FALSE	1,603	0,057	FALSE	0
DSSD61	0,025	0,933	FALSE				0,062	0,863	FALSE	0
Dadabacteriales	-0,405	0,437	FALSE				0,490	0,174	FALSE	0
Fimbriiglobus	-0,391	0,295	FALSE				-0,281	0,354	FALSE	0
Fluviicola	0,065	0,920	FALSE	-0,434	0,415	FALSE	0,228	0,838	FALSE	0
Gemmata	-0,127	0,788	FALSE							0
Gemmobacter	-0,251	0,630	FALSE	-0,571	0,170	FALSE	0,476	0,284	FALSE	0
Halomonas	0,598	0,166	FALSE	0,089	0,811	FALSE	-0,281	0,472	FALSE	0
Hydrogenophaga	0,259	0,626	FALSE	-0,330	0,453	FALSE				0
Hyphomicrobium	-0,490	0,295	FALSE	-0,402	0,258	FALSE	-0,213	0,665	FALSE	0
IMCC26207	-0,070	0,864	FALSE				0,082	0,863	FALSE	0
Isosphaera	-0,694	0,096	FALSE							0
Luteolibacter	0,729	0,096	FALSE	0,436	0,243	FALSE	0,321	0,365	FALSE	0
Lysobacter	0,831	0,096	FALSE	0,607	0,165	FALSE	-0,148	0,807	FALSE	0
Mycobacterium	0,127	0,788	FALSE	-0,181	0,602	FALSE	-0,175	0,665	FALSE	0
Neochlamydia	-0,260	0,651	FALSE	0,188	0,638	FALSE				0
Nitrospira	0,421	0,387	FALSE	0,077	0,838	FALSE	0,552	0,298	FALSE	0
Nordella	0,230	0,626	FALSE				-0,042	0,960	FALSE	0
OM190	0,483	0,212	FALSE	-0,290	0,445	FALSE	0,048	0,958	FALSE	0
Obscuribacteraceae	0,370	0,371	FALSE				-0,276	0,382	FALSE	0

Pannonibacter	0,567	0,170	FALSE				0,533	0,298	FALSE	0
Paracoccus	0,365	0,369	FALSE				0,002	0,994	FALSE	0
Pelomonas	0,266	0,522	FALSE							0
Phreatobacter	0,032	0,933	FALSE	-0,481	0,188	FALSE	-0,105	0,838	FALSE	0
Planctopirus	-0,116	0,788	FALSE				0,007	0,994	FALSE	0
Pseudoxanthomonas	-0,028	0,933	FALSE	0,296	0,484	FALSE	0,333	0,580	FALSE	0
Reyranella	0,550	0,254	FALSE	0,647	0,078	FALSE	-0,168	0,838	FALSE	0
Rheinheimera	0,074	0,901	FALSE	0,286	0,486	FALSE	0,551	0,148	FALSE	0
Rhodobacter	0,556	0,218	FALSE	0,270	0,484	FALSE	0,564	0,253	FALSE	0
SH3-11	0,326	0,461	FALSE	0,095	0,807	FALSE	0,110	0,838	FALSE	0
Saccharimonadales	0,582	0,197	FALSE	-0,138	0,764	FALSE	0,717	0,155	FALSE	0
Staphylococcus	0,453	0,326	FALSE	0,249	0,636	FALSE	0,643	0,472	FALSE	0
Streptococcus	0,294	0,626	FALSE	-0,105	0,838	FALSE	1,039	0,174	FALSE	0
Unknown_Family	-0,091	0,840	FALSE	-0,447	0,243	FALSE	0,008	0,994	FALSE	0
Vibrio	1,538	0,084	FALSE	1,065	0,078	FALSE	0,492	0,411	FALSE	0
Cetobacterium				0,412	0,392	FALSE	0,382	0,376	FALSE	0
Enhydrobacter				0,284	0,492	FALSE	-0,094	0,863	FALSE	0
Mitochondria				0,487	0,236	FALSE	-0,032	0,986	FALSE	0
SAR324_clade(Marine_group_B)				-0,307	0,392	FALSE				0
env.OPS_17				0,191	0,621	FALSE	-0,147	0,694	FALSE	0
0319-6G20							0,484	0,411	FALSE	0
Acidibacter							-0,090	0,838	FALSE	0
Alcaligenes							0,778	0,148	FALSE	0
Anaerococcus							0,660	0,155	FALSE	0
Aquicella							-0,138	0,766	FALSE	0
Bacillus							0,291	0,665	FALSE	0

Brevundimonas							0,156	0,799	FALSE	0
Bryobacter							-0,075	0,863	FALSE	0
Candidatus_Megaira							-0,082	0,854	FALSE	0
Cellvibrio							-0,452	0,284	FALSE	0
Chryseobacterium							-0,436	0,201	FALSE	0
Chthoniobacter							0,051	0,926	FALSE	0
Crenobacter							0,107	0,838	FALSE	0
Devosia							-0,562	0,148	FALSE	0
Dolosigranulum							0,602	0,105	FALSE	0
Fimbriimonadaceae							0,899	0,148	FALSE	0
Flavobacterium							0,175	0,668	FALSE	0
Friedmanniella							0,635	0,105	FALSE	0
Haemophilus							0,119	0,807	FALSE	0
Helcococcus							-0,147	0,766	FALSE	0
Hymenobacter							0,733	0,271	FALSE	0
JG30-KF-CM45							0,089	0,863	FALSE	0
Kapabacteriales							0,005	0,994	FALSE	0
Lactobacillus							0,002	0,994	FALSE	0
Lawsonella							-0,021	0,986	FALSE	0
Legionella							-0,647	0,271	FALSE	0
Marmoricola							0,750	0,105	FALSE	0
Massilia							1,020	0,148	FALSE	0
Methylobacterium-Methylorubrum							-0,267	0,668	FALSE	0
Micrococcus							0,124	0,838	FALSE	0
NB1-j							0,008	0,994	FALSE	0

NS11-12_marine_group							-0,471	0,342	FALSE	0
Negativicoccus							0,140	0,764	FALSE	0
Neisseria							0,029	0,986	FALSE	0
Nocardioides							0,283	0,411	FALSE	0
Nosocomiicoccus							-0,653	0,123	FALSE	0
Pedobacter							-0,398	0,253	FALSE	0
Pedomicrobium							-0,091	0,856	FALSE	0
Prevotella							0,051	0,917	FALSE	0
Psychrobacter							0,342	0,354	FALSE	0
RBG-13-54-9							0,062	0,863	FALSE	0
Roseomonas							0,592	0,217	FALSE	0
Rubellimicrobium							0,024	0,994	FALSE	0
SH-PL14							0,002	0,994	FALSE	0
Skermanella							0,546	0,472	FALSE	0
Sphingomonas							0,333	0,580	FALSE	0
Subgroup_2							-0,144	0,799	FALSE	0
UBA12409							-0,026	0,978	FALSE	0
Vicinamibacteraceae							0,107	0,838	FALSE	0
Vogesella							0,071	0,949	FALSE	0
WPS-2							0,085	0,863	FALSE	0
mle1-8							-0,159	0,665	FALSE	0

Figure S1. Volcano plots showing the ANCOM-BC2 differential abundance analysis for the Illumina V3–V4 (A) and ONT V3–V4 (B) datasets.

(A)

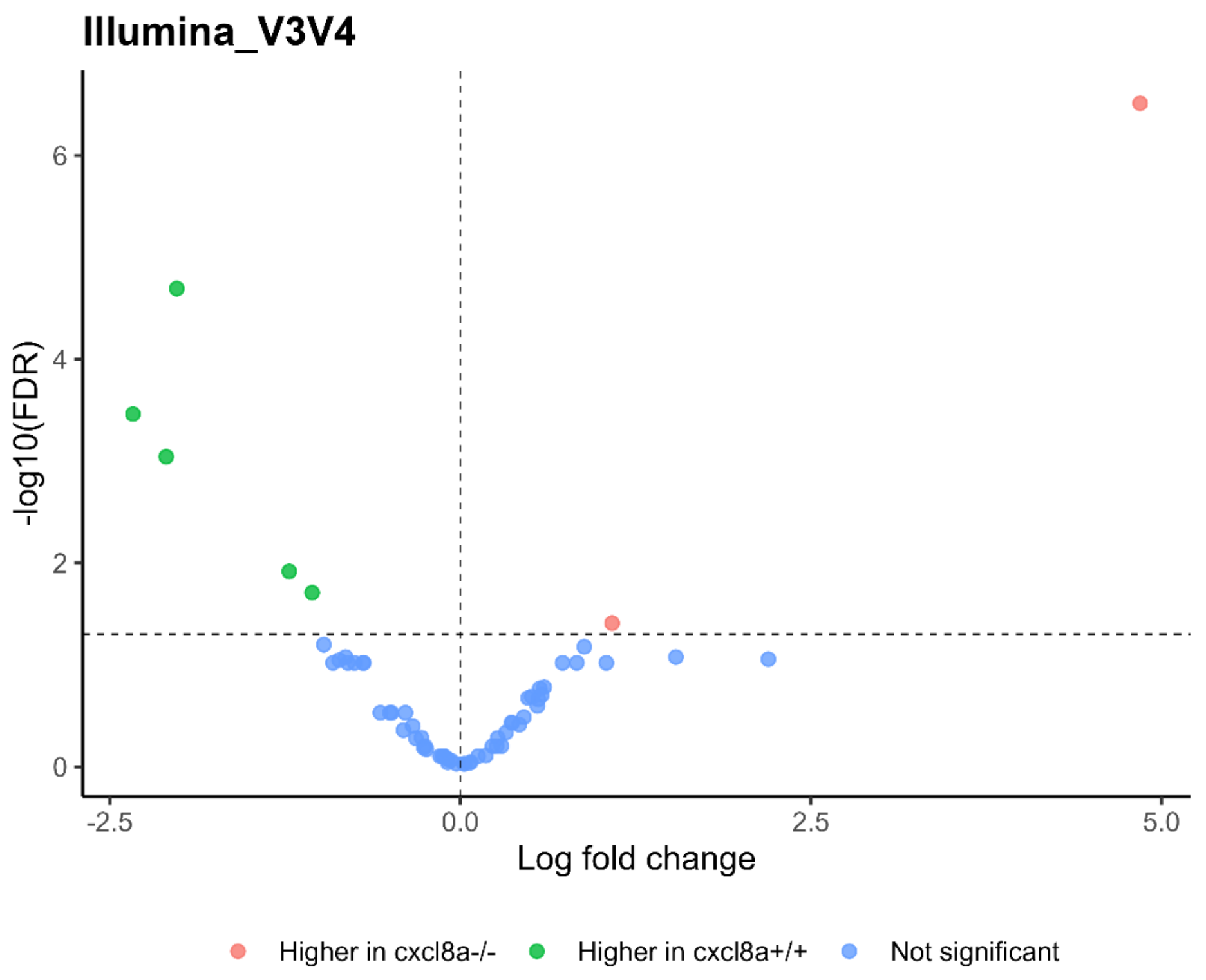

(B)

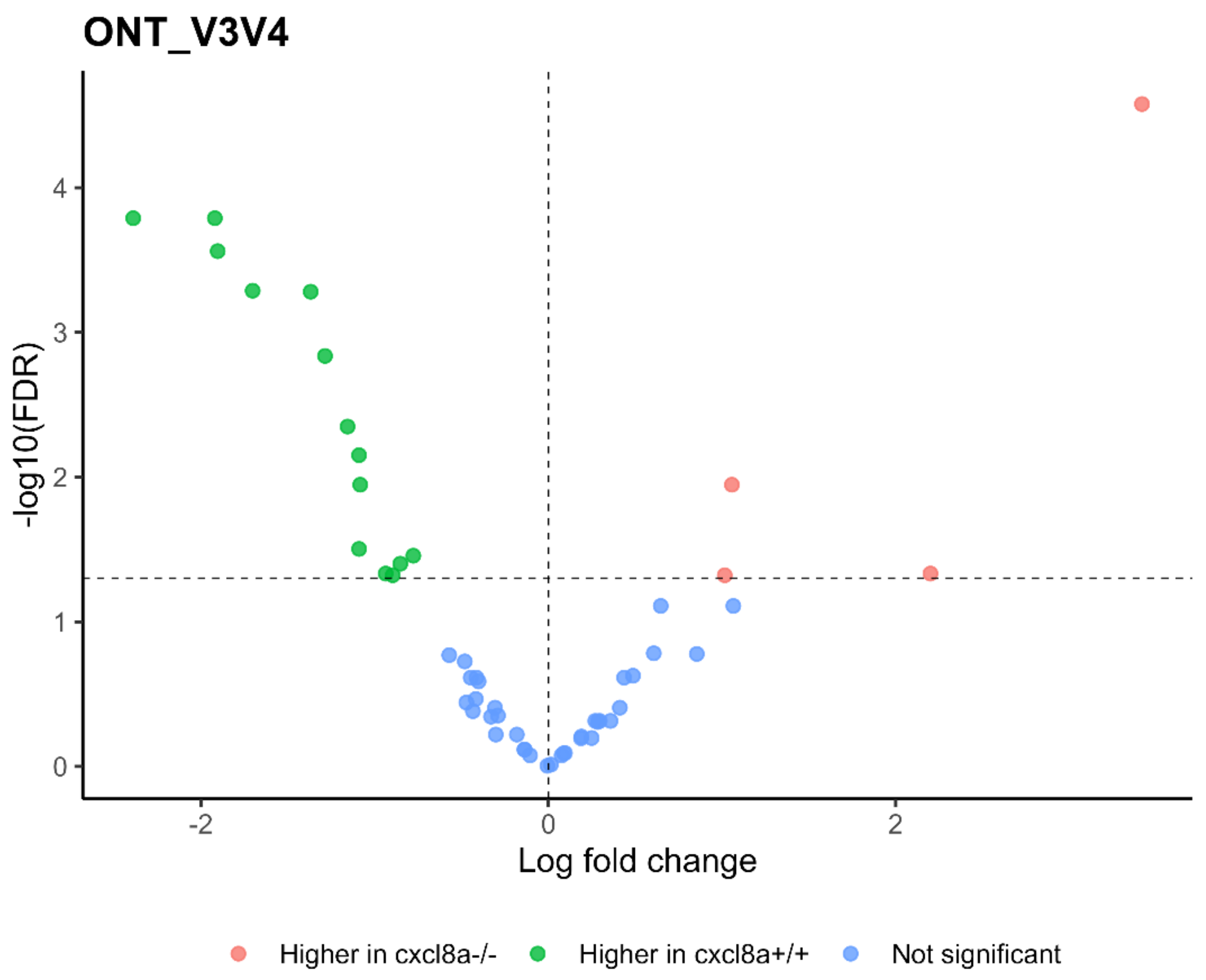

Figure S2. Sankey diagram showing the hierarchical taxonomic composition of the combined ONT V1–V9 dataset inferred using the EPI2ME 16S workflow. Branch widths are proportional to the cumulative number of classified reads.

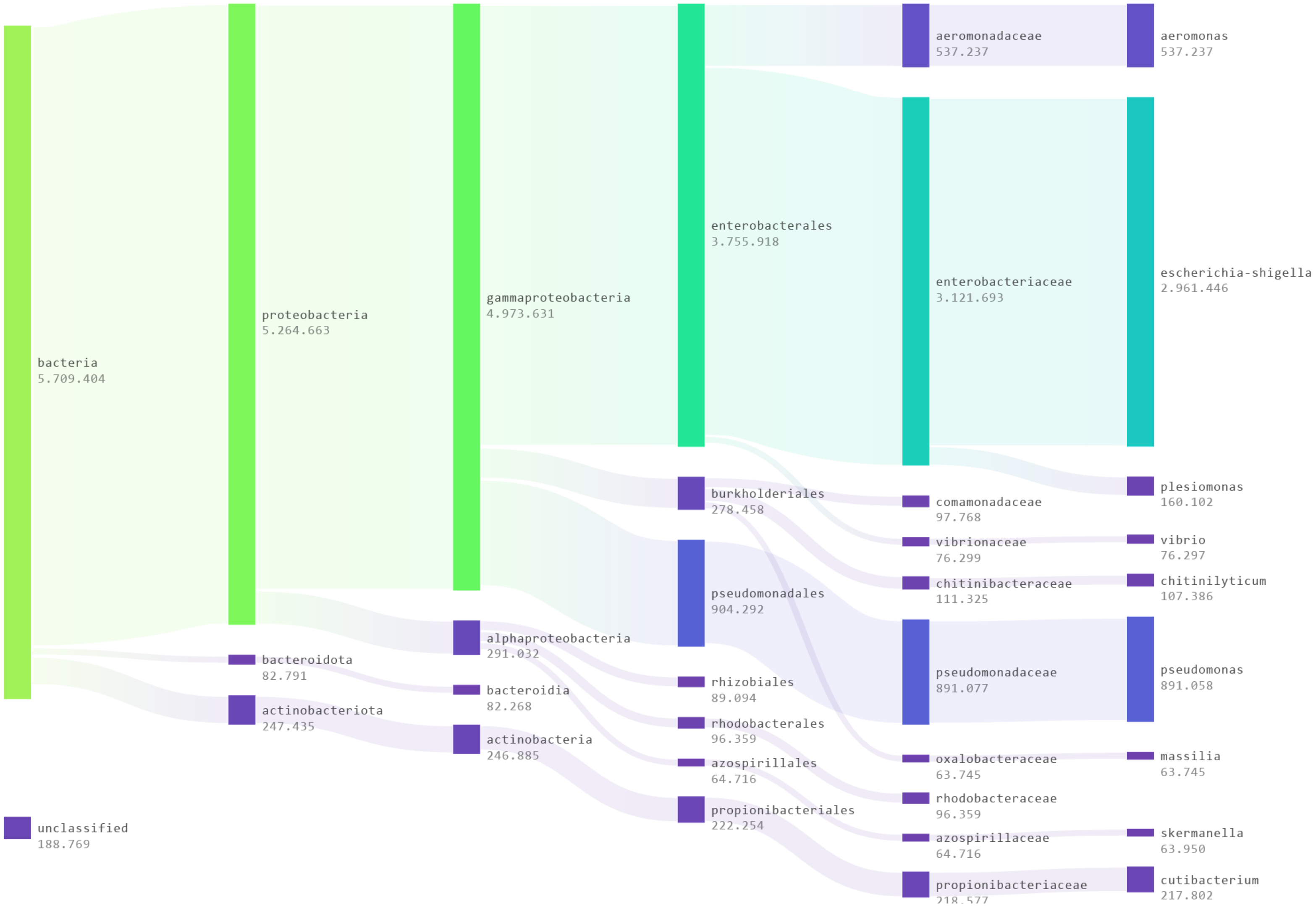

Figure S3 Rarefaction analysis of the Illumina V3–V4 and ONT datasets. (a) Mean rarefaction curves (± SEM) comparing Illumina V3–V4 and ONT V3–V4 datasets. The dashed vertical line indicates the rarefaction depth of 50,000 reads used for downstream analyses. (b) Mean rarefaction curve (± SEM) for the ONT V1–V9 dataset

(a)

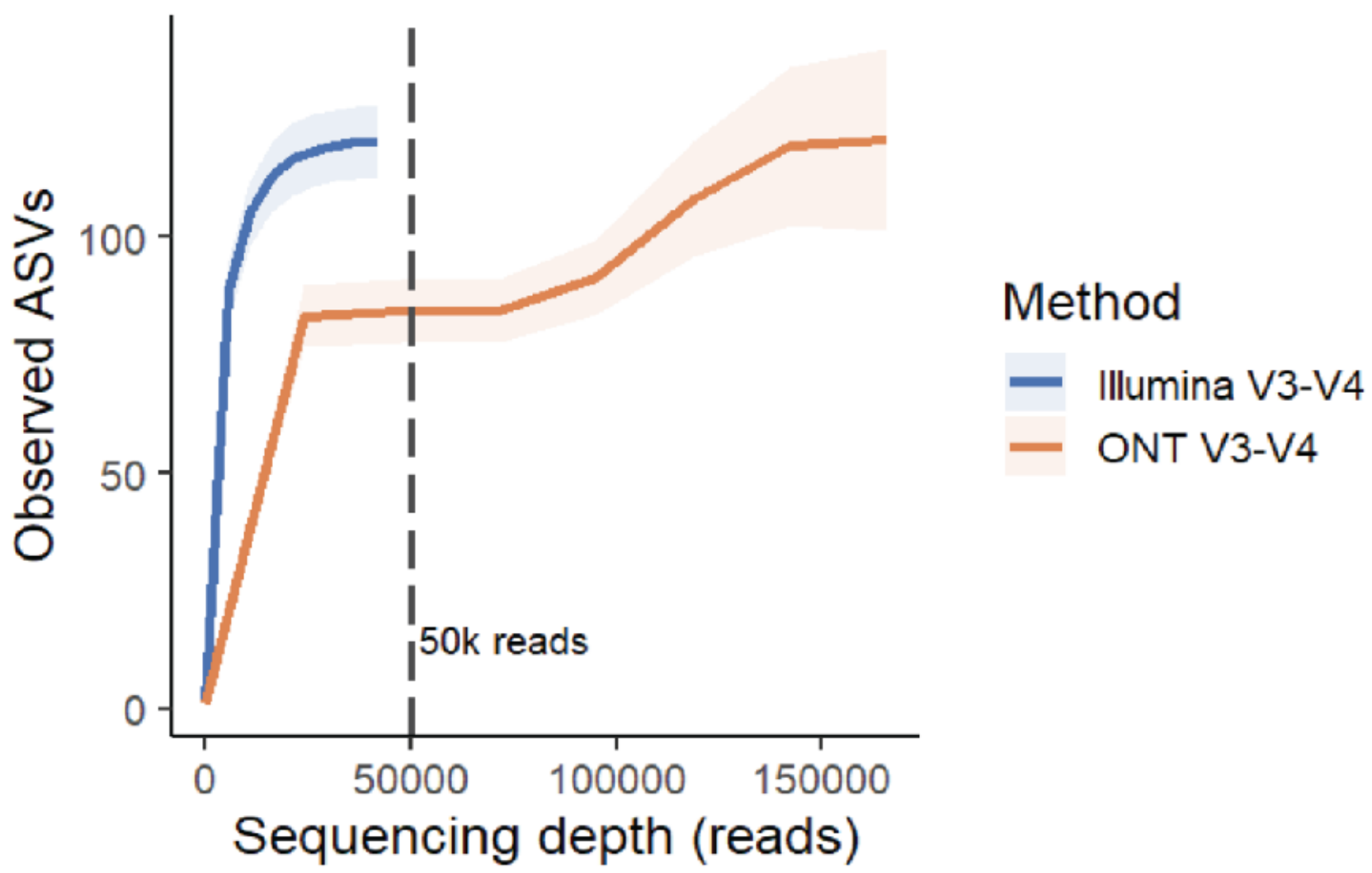

(b)

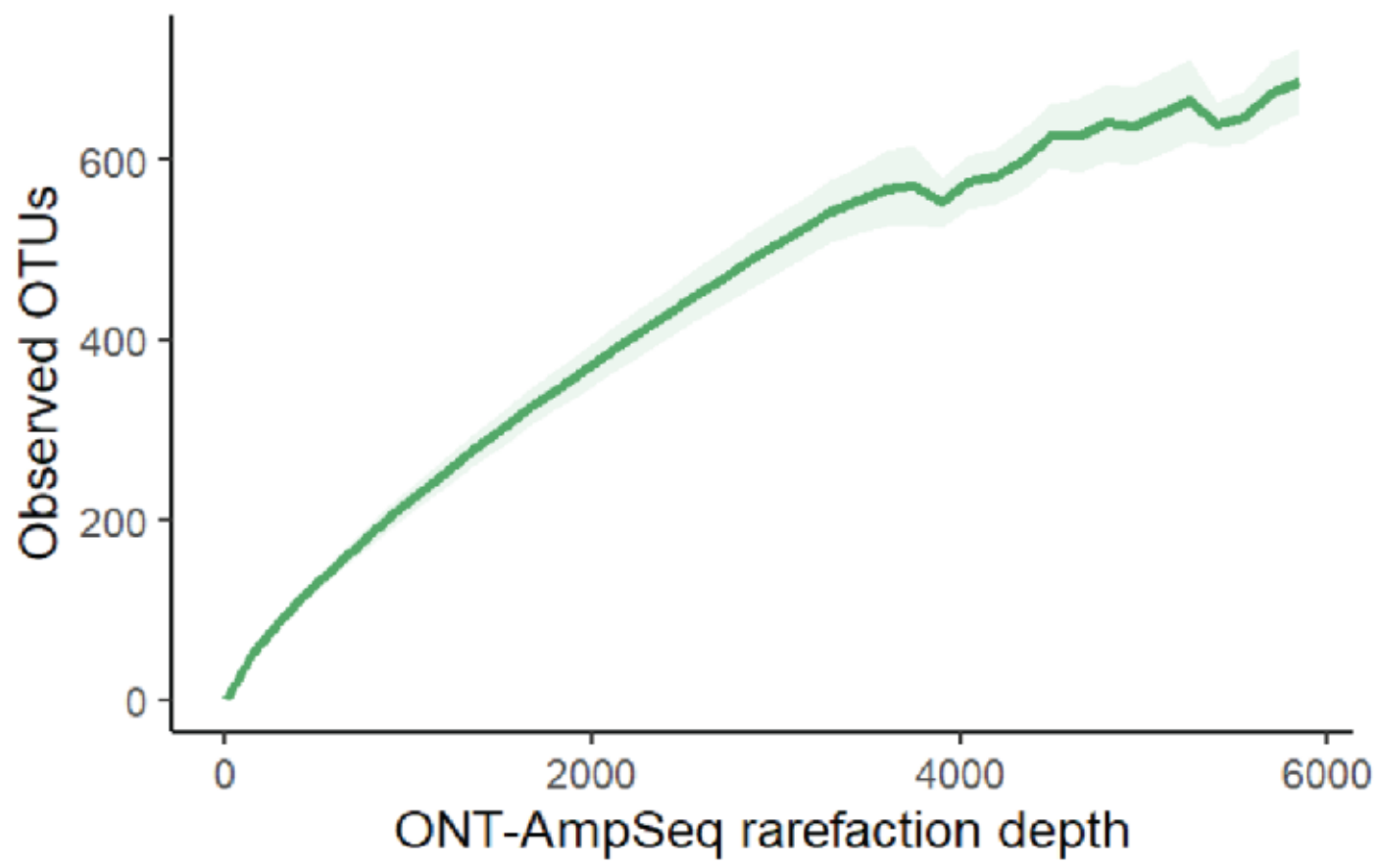

Supplement: Supplementary file 1 [file mgen-12-01828-s001.pdf]
